# Breeding of High-Polysaccharide-Producing *Volvariella volvacea* Strains Based on Genome Shuffling Technology

**DOI:** 10.3390/jof11080591

**Published:** 2025-08-14

**Authors:** Lihui Liang, Qihang Su, Yawei Wang, Peichen Du, Suzhen Zhao, Huanjie Zhang, Xiaofeng Gao

**Affiliations:** 1 National R & D Center for Edible Fungus Processing Technology, Henan University, Kaifeng 475004, China15737858141@163.com (Q.S.); wangyawei2024@126.com (Y.W.); 17337858328@163.com (P.D.); zhaoshuzhen2023@163.com (S.Z.); zhj1999521@163.com (H.Z.); 2 College of Agriculture, Henan University, Kaifeng 475004, China; 3 Henan Dabieshan National Field Observation and Research Station of Forest Ecosystem, Henan University, Kaifeng 475004, China; 4 School of Life Sciences, Henan University, Kaifeng 475004, China

**Keywords:** *Volvariella volvacea*, ARTP, genome shuffling, high polysaccharide yield, extracellular polysaccharides

## Abstract

*Volvariella volvacea*, a fungal species of *Volvariella* within the Pluteaceae family, is predominantly cultivated in southern China. Polysaccharides, the primary bioactive constituents of *V. volvacea*, exhibit diverse pharmacological activities. However, current cultivation practices face challenges due to the genetic heterogeneity of strains, leading to inconsistent content and compositional variability of polysaccharides and other functional components. ARTP, denoting atmospheric and room-temperature plasma, is a technology capable of generating plasma jets at ambient pressure with temperatures ranging from 25 to 40 °C. These jets feature high concentrations of highly reactive species, including but not limited to excited-state helium atoms, oxygen atoms, nitrogen atoms, and OH radicals. This study aims to develop high-yielding exopolysaccharide (EPS) strains through integrated ARTP mutagenesis and genome shuffling, thereby overcoming current cultivation bottlenecks. ARTP mutagenesis and genome shuffling significantly boosted EPS production in *V. volvacea*. ARTP generated nine stable mutants with >20% higher EPS yields. Subsequent genome shuffling (three rounds of protoplast fusion) produced the hybrid strain SL212, which achieved 46.85 g/L of EPS, an 111.67% increase over that of the parent strain under identical conditions. Metabolomics and transcriptomics analyses revealed that differential metabolites and genes were mainly enriched in galactose metabolism, ABC transporter pathways, and the tricarboxylic acid cycle. These pathways enhance monosaccharide biosynthesis and generate ATP, providing both precursors and energy for polysaccharide polymerization, thereby driving EPS overproduction. Preliminary mechanistic analysis identified the key contributing factors driving the elevated polysaccharide biosynthesis.

## 1. Introduction

*Volvariella volvacea* (Bull.) Singer, commonly known as orchid mushroom, hemp mushroom, or Chinese mushroom, is a typical species of tropical and subtropical edible fungi [1,2]. It is primarily cultivated in Hunan, Taiwan, Guangxi, and Guangdong. *V. volvacea* is nutritionally dynamic and rich in both macronutrients and micronutrients, with its composition varying significantly across developmental stages [3,4]. Notably, it contains a complete amino acid profile. Additionally, potassium and magnesium concentrations are elevated during the button stage relative to the other growth phases [5]. Notably, this species has the highest vitamin D content among edible mushrooms, significantly enhancing its nutritional value [6]. Furthermore, carbohydrates constitute 50–65% of the fruiting body’s dry mass, with a progressive increase observed during maturation [5]. The diverse nutrient profile of *V. volvacea* fulfills daily dietary requirements, underscoring its potential as a candidate for functional food development. In addition, polysaccharides are the main functional components of *V. volvacea.*

Polysaccharides are natural polymers composed of over ten monosaccharide units linked via β-(1→3) or β-(1→6) glycosidic bonds [7]. They are ubiquitous in plants, animals, and microorganisms [8,9,10,11]. *V. volvacea* polysaccharides possess pharmacological activities such as anticancer [12], antioxidant [13,14,15], antimicrobial [16], immunomodulatory [17], anti-inflammatory [18], hypoglycemic [19,20], and hypolipidemic effects [21] and mitigate carcinogenicity [22], highlighting their dual role as exceptional functional food ingredients and promising candidates for pharmaceutical applications.

Atmospheric and room-temperature plasma (ARTP) mutagenesis employs reactive species (e.g., helium, nitrogen) in excited states to interact with cellular components, thereby altering membrane permeability, disrupting cell wall integrity, and inducing DNA lesions [23]. This damage activates microbial SOS repair systems to restore genomic stability [24,25,26]. Distinguished by its elevated mutagenic efficiency, user-friendly protocol, and environmentally benign nature, ARTP has been successfully applied to bacteria [27,28,29], fungi [30,31,32], and microalgae [33,33], demonstrating consistent efficacy in enhancing metabolite yields.

Genome shuffling, a mutagenic strategy that alters the genomic content or gene arrangement through recursive protoplast fusion, integrates traditional mutagenesis with cell engineering. This technique enables genome-wide recombination across multiple parental strains, selects phenotypically superior mutants, and significantly enhances positive mutation rates, making it a transformative method for rapid microbial genome optimization [34,35,36]. First conceptualized by Zhang et al. in 2002 [37], genome shuffling has expanded mutagenesis from single-parent to multi-parent frameworks by merging classical breeding with whole-genome engineering. Its applications include bacterial and fungal strain improvements, enhancing substrate utilization efficiency, boosting metabolite yields, improving environmental stress tolerance, and refining metabolic pathways [34,38,38,39,40].

This study addresses the scarcity of parental resources for genome shuffling by constructing a highly diverse mutant library via ARTP, achieving a “high-efficiency mutation-precise aggregation” synergy. This breakthrough overcomes the limitations of single technologies, enabling rapid aggregation of multiple positive mutations to optimize the complex metabolic network underlying polysaccharide synthesis in *V. volvacea*. However, as a filamentous fungus, *V. volvacea* presents certain limitations in this approach: genome shuffling entails a heavy workload in screening high-yield strains. It may be accompanied by undesirable mutations that affect other traits of the strains.

## 2. Materials and Methods

### 2.1. Microorganism and Culture Media

#### 2.1.1. Original Microorganism

*V. volvacea* and a fusion strain were used, stored at the National R & D Center for Edible Fungus Processing Technology of Henan University.

#### 2.1.2. Culture Media and Culture Conditions

The laboratory conducted preliminary experiments, The medium composition (per L) was 200 g of boiled potato residue (filtered), 55.0 g of maltose, 1.5 g of MgSO_4_, 3.0 g of K_2_HPO_4_, and 10 mg of vitamin B_1_. Sterilization at 121 °C for 20 min under high pressure was performed.

The culture parameters were an initial pH of 5.5, an agitation speed of 150 rpm, and 5-day fermentation.

#### 2.1.3. Solution Preparation

We utilized PDA regeneration medium comprising (per L) 200 g of boiled potatoes (filtered), 20 g of glucose, and 20 g of agar dissolved in 0.6 M mannitol, and we performed sterilization at 121 °C for 20 min under high pressure; we used 2% lysozyme solution comprising 0.02 g lysozyme dissolved in 1 mL of 0.6 M mannitol and filter-sterilized using 0.22 μm disposable sterile filters (prepared fresh before use); and we used 7.00% snailase solution comprising 0.070 g of snailase dissolved in 1 mL of 0.6 M mannitol and filter-sterilized using 0.22 μm filters. The fusion agent was 0.4 g/mL polyethylene glycol (PEG) prepared by dissolving 40 g of PEG 6000 in 0.6 M mannitol, supplemented with 10 mL of 0.1 M CaCl_2_. The pH was adjusted to 8.0 and the final volume was adjusted to 100 mL prior to autoclave sterilization.

### 2.2. Determination of EPS Content

The total soluble sugar content was assayed using the Soluble Sugar Content Assay Kit (Solarbio, Beijing, China) via the anthrone-sulfuric acid method [41]. This protocol relies on the dehydration of carbohydrates in concentrated sulfuric acid to form furfural derivatives, which subsequently react with anthrone to produce a bluish-green chromogenic complex that can be measured at 620 nm.

Reducing sugars were quantified using a Reducing Sugar Assay Kit (Solarbio) employing 3,5-dinitrosalicylic acid [42]. Under alkaline conditions, the reducing sugars reduce 3,5-dinitrosalicylic acid to 3-amino-5-nitrosalicylic acid during heating, generating a brick-red compound with a characteristic absorption peak at 540 nm.

### 2.3. Determination of Dosage of the ARTP Mutagen

Under aseptic conditions, 20 μL of the prepared cell suspension of *V. volvacea* was transferred to a glass slide and subjected to ARTP irradiation for 0, 10, 20, 30, 40, 50, or 60 s. Following mutagenesis, the cell suspensions were serially diluted and plated on PDA medium alongside non-irradiated controls. After 5 days of incubation at 32 °C, colony counts were recorded for each treatment duration. The lethality rates for different treatment times were calculated (%) as follows: [(U − T)/U]*100, where U is the total colony count of the sample after treatment and T is the total colony count after treatment with ARTP.

### 2.4. Screening of Mutant Strains

For primary screening, a dual-culture plate assay was performed by inoculating the parental and mutant strains at opposite ends of PDA plates [43]. After incubation at 32 °C for 5 days, distinct antagonistic reactions between the different strains were visually assessed. Mutant strains exhibiting clear inhibition zones were documented and preserved for subsequent screening.

For secondary screening, the selected mutants demonstrating significant antagonistic activity were subjected to shake-flask fermentation using the optimized liquid culture conditions [44]. Following cultivation, EPS production was quantified to identify strains with statistically superior yields compared with the original strain. Five successive subcultures were conducted to verify genetic stability before the final preservation on agar slants for future applications.

### 2.5. Protoplast Fusion

*V. volvacea* mycelia were aseptically inoculated into the liquid medium and incubated statically at 33 °C for 5 days. After cultivation, the hyphal biomass was harvested by filtration through a quadruple-layer sterilized gauze, followed by three rinses with sterile deionized water to remove the residual medium. Surface moisture was gently absorbed using sterile filter paper.

The mycelial pellets were resuspended in an equal volume of dual-enzyme solution comprising 2.0% (*w*/*v*) lysozyme and 7.0% (*w*/*v*) snailase and subjected to enzymatic digestion at 32 °C with orbital shaking (100 rpm) for 3 h. The resulting lysate was sequentially filtered through a G3 sintered glass funnel to remove cellular debris and then centrifuged (4000 rpm, 4 °C, 10 min) to pellet protoplasts. The pelleted protoplasts were washed twice with 0.6 M mannitol solution to eliminate residual enzymes and finally resuspended in fresh 0.6 M mannitol. Protoplast density was quantified via hemocytometer and adjusted to 10^3^/mL using a sterile mannitol solution.

### 2.6. Inactivation of Parental Genes

Heat inactivation: Under aseptic conditions, protoplast suspensions were aliquoted into 1.5 mL sterile centrifuge tubes, sealed with parafilm, and immersed in water baths maintained at 35 °C, 40 °C, 45 °C, and 50 °C. Isothermal treatments were performed for durations of 5, 10, 15, 20, and 25 min at each temperature. Post-inactivation, 100 μL aliquots were uniformly plated on PDA regeneration medium. Cultures were incubated at 32 °C under dark conditions for 5 days. Untreated suspensions served as controls, with triplicate samples per experimental group. Inactivation rates were calculated using Equation (1).

Ultraviolet inactivation: Under aseptic conditions, protoplast suspensions were dispensed into sterile Petri dishes. These dishes were positioned 20 cm beneath a pre-warmed 19 W UV lamp for irradiation exposure. Suspensions underwent UV treatment for durations of 0, 1, 5, 10, 15, 20, and 25 min. Post-exposure, 100 μL aliquots were uniformly plated onto PDA regeneration medium. Plates were dark-incubated at 32 °C for 5 days. Untreated suspensions served as controls, with triplicate replicates per treatment group. Inactivation rates were calculated according to Equation (1).

(1) $$P=\frac{A-B}{A}$$

*P*: plasmid inactivation rate; *A*: the number of colonies before inactivation; *B*: the number of colonies after inactivation.

### 2.7. Genome Shuffling

Protoplast suspensions derived from the mutagenized strains were prepared by combining equal volumes (1:1) of the differentially inactivated parental suspensions in sterile centrifuge tubes. Following centrifugation (8000 rpm, 10 min), the supernatants were aspirated to harvest the hybrid protoplast aggregates. The protoplast-PEG 6000 fusion complex was established by thorough mixing with 0.6 M mannitol solution containing 40% (*w*/*v*) of the PEG 6000 fusogenic agent. This mixture was subjected to fusion induction by incubation in a 35 °C water bath for 30 min with periodic gentle agitation. Post-fusion treatment, residual PEG was removed via three washes using 0.6 M mannitol. The purified protoplasts were reconstituted in fresh 0.6 M mannitol solution, and 100 μL aliquots were aseptically spread on PDA regeneration medium supplemented with osmotic stabilizers. The plates were incubated at 32 °C for 5 days to monitor fusion-derived colony formation.

Primary recombinant strains (designated the A1 generation) demonstrating enhanced EPS biosynthesis and distinct antagonism against the parental *V. volvacea* strain were selected as the progenitor pool for iterative genome shuffling. The A1 recombinants underwent secondary genome shuffling through protoplast fusion, yielding a fusion-derived A2 progeny that retained the two selection criteria of amplified antagonistic activity and superior EPS productivity relative to the ancestral strain. This recursive shuffling protocol was repeated three times. Successive recombinant cohorts from each shuffling iteration were assigned sequential identifiers (SL1, SL2, SL3, etc.), maintaining traceability throughout the multigenerational screening.

### 2.8. Transcriptomics and Metabolomics Analysis

#### 2.8.1. Metabolomic Analysis

A fresh, comprehensive PDA medium was prepared and sterilized. Spores of *V. volvacea* and SL212 were inoculated into 50 mL of liquid medium and cultured at 150 rpm for 5 days, with six biological replicates per group. After freezing in liquid nitrogen, the samples were stored at −80 °C.

Metabolic differences between the parental *V. volvacea* and hybrid strain SL212 were profiled using LC-MS/MS. Following chromatographic separation on an Agilent 1290 Infinity LC UHPLC system (Agilent, Santa Clara, CA, USA), samples underwent mass spectrometric characterization using a TripleTOF 6600 instrument (SCIEX, Marlborough, MA, USA). Detection was performed in both positive and negative electrospray ionization (ESI) modes with the following source parameters: Nebulizer Gas (Gas1): 60 psi; Heater Gas (Gas2): 60 psi; Curtain Gas (CUR): 30 psi; Source Temperature: 600 °C; Ion Spray Voltage: ±5500 V (positive/negative modes)

#### 2.8.2. Transcriptomic Analysis

Mycelia scraped from the comprehensive PDA medium were collected in 2.0 mL centrifuge tubes and stored. Each group comprised three biological replicates. After freezing in liquid nitrogen, the samples were stored at −80 °C.

Total RNA was extracted from the samples. Upon verification of RNA integrity, target fragments were isolated and amplified via PCR for library construction. High-quality sequences were assembled into unigenes, followed by bioinformatic analysis. Raw reads underwent quality filtering to remove adapter-contaminated sequences, low-quality bases (Q-score < 20), and reads containing ambiguous bases (N > 5%), yielding final clean reads for downstream analysis.

The *V. volvacea* strain was designated as the CG group and the SL212 strain as the EG.

### 2.9. Real-Time Quantitative Polymerase Chain Reaction (RT-qPCR)

Total RNA was extracted from the samples. Reverse transcription of extracted RNA was performed using the HiScript 1st Strand cDNA Synthesis Kit (Vazyme, Nanjing, China). Subsequent quantitative PCR employed SYBR Green I detection chemistry (AceQ qPCR SYBR Green Master Mix, ROX-free). The primer sequences can be found in Supplementary Information Table S1.

### 2.10. Statistical Analysis

Statistical analysis was performed using Origin. All data are expressed as the mean ± standard deviation, with significance determined by one-way ANOVA (*p* < 0.05). For metabolomics, Raw data were converted to .mzXML format via ProteoWizard. XCMS was employed for peak alignment, retention time correction, peak area extraction, metabolite structural annotation, data preprocessing, quality control assessment, and statistical analysis. For transcriptomics, reads were aligned to the reference genome using HISAT2 with its optimized BWT algorithm. Gene-level quantification was performed with featureCounts (v2.0.3), calculating FPKM values.

## 3. Results

### 3.1. Effect of ARTP Mutagenesis Time on Lethality Rate

**Figure 1:**
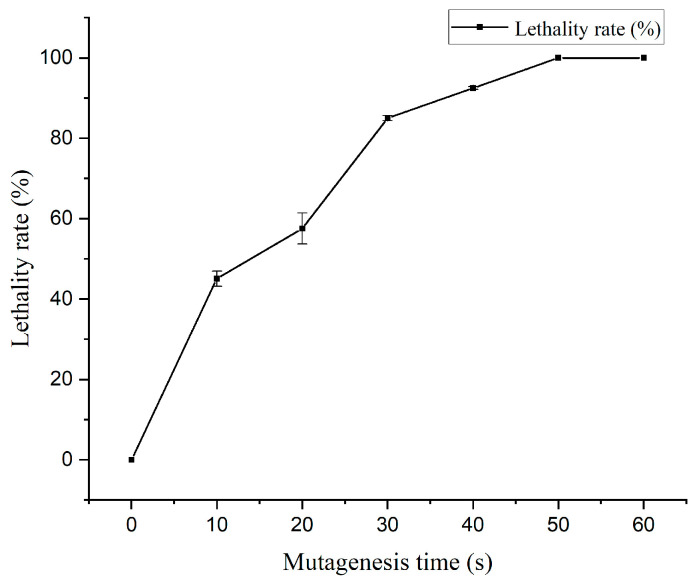
Effect of ARTP mutagenesis time on lethality rate. Each treatment contained three replicates.

The ARTP-induced lethality curve for *V. volvacea* is shown in Figure 1. Within 60 s of ARTP treatment, fungal lethality progressively increased with prolonged exposure time, reaching 84.98% at 30 s and 100% at 50 s. A previous study [45] documented that mutagenic efficiency is maximized when lethality ranges between 80% and 90% because this interval promotes abundant random positive mutations. Excessive lethality significantly increases the probability of detrimental negative mutations, which complicates the screening for beneficial mutants. Consequently, 30 s was identified as the optimal ARTP mutagenesis duration for *V. volvacea*, balancing mutation diversity and viability retention.

### 3.2. Screening of Mutagenic Strains

Following ARTP mutagenesis, 135 mutant strains (designated HY1–HY135) were isolated and cryopreserved in PDA slants for subsequent screening.

#### 3.2.1. Primary Screening of Mutagenized Strains

A plate confrontation assay between the parental *V. volvacea* and the 135 ARTP-mutagenized derivatives identified 19 strains with distinct antagonistic responses (mutation frequency: 14.07%). The partial antagonistic diagram can be found in Supplementary Information Figure S1.

#### 3.2.2. Secondary Screening of Mutagenized Strains

The 19 candidate mutants were subjected to secondary shake-flask screening with five serial subculturing cycles (Supplementary Information Table S2). Four, two, and three of the mutants displayed ≥50%, 30%, and 20% higher EPS yields than the parental strain, respectively. Strain HY115 showed peak EPS productivity with a 71.52% increase. After sub-culturing, EPS biosynthesis retained functional stability across all lineages, qualifying these strains for downstream applications.

### 3.3. Genome Shuffling

Nine mutagenized strains (HY24, HY31, HY4, HY67, HY112, HY73, HY123, HY103, and HY115) demonstrating ≥30% enhanced yield of EPS were selected as the primary parental pool. First-round genome shuffling via dual-parent chemical inactivation-mediated protoplast fusion yielded 19 fusants, including 5 strains with significant EPS productivity gains. These 5 elite fusants formed the second parental pool, generating 26 fusants through subsequent shuffling, 7 of which exhibited enhanced biosynthesis. The tertiary shuffling cycle using these 7 strains produced 23 fusants, of which 9 demonstrated superior EPS yields. All shuffled strains were systematically designated SL1, SL2……

After three rounds of genome shuffling, nine fusants with significantly enhanced polysaccharide production compared to the original strain were selected. The EPS yields of different strains are shown in Supplementary Information Table S3; each group is set up with three parallel sections. Strain SL212 displayed the greatest increase in polysaccharide production, reaching 46.85 g/L, representing a 111.67% improvement over the original strain.

To assess the genetic stability of SL212 in terms of EPS production, the strain was subjected to five successive subcultures and shake flask cultivation. As shown in Table 1, no significant fluctuations in mycelial growth or EPS production were observed after five generations. Therefore, strain SL212 was selected for further experiments.

### 3.4. Comparative Metabolomics Analysis of V. volvacea

Liquid chromatography–tandem mass spectrometry was used to analyze 12 samples from the original strain of *V. volvacea* and the fusion strain SL212 in both positive and negative ion modes. The raw data underwent initial processing, including missing-value filtration, simulation, quality control validation, normalization, and data transformation. In the positive ion mode, 1083 components were identified. In the negative ion mode, 464 components were detected. Among these, 712 and 412 metabolites in the positive and negative ion modes, respectively, were successfully annotated in the Kyoto Encyclopedia of Genes and Genomes (KEGG) database. The metabolite components annotated by KEGG in the positive and negative ion modes are shown in Supplementary Information Tables S4 and S5.

#### 3.4.1. Metabolomics Multivariate Statistical Analysis

Multivariate statistical analysis was performed to identify potential differential metabolites between the original *V. volvacea* strain (CG group) and the fused strain SL212 (EG group). Principal component (PC) analysis (PCA) score plots were generated to visualize the distribution of samples in the reduced-dimensional PC space, reflecting similarities and differences among groups. As shown in Supplementary Information Figure S2, a clear separation between the CG and EG groups was observed in both positive and negative ion modes, indicating significant metabolic differences. In the positive ion mode, PC1 accounted for 50.5% of the variance on the x-axis, while PC2 explained 6% on the y-axis. Similarly, in the negative ion mode, PC1 contributed 49.1% (x-axis), and PC2 represented 5.7% (y-axis). Minimal intragroup variability and distinct intergroup separation confirmed the reliability of the data, supporting its suitability for subsequent analysis.

Unlike PCA, Partial Least Squares Discriminant Analysis (PLS-DA) reduces dimensionality by extracting latent variables, maximizing the explanation of relationships between variables (X) and class labels (Y). Additionally, PLS-DA incorporates sample category information during dimensionality reduction, making it more suitable for classification and discriminant analyses. Building on the PCA results, PLS-DA was applied to the metabolomic data from the CG and EG in both ion modes. Q^2^, a predictive metric of the PLS-DA model, represents the proportion of response variables explainable in cross-validation, with Q^2^ > 0.5 indicating a robust predictive capability. The results demonstrated Q^2^ values of 0.992 (positive ion mode) and 0.9 (negative ion mode), confirming minimal false positives and high model reliability. As shown in Supplementary Information Figure S3, samples within the CG and EG clustered tightly, while intergroup samples were distinctly separated, further validating the significant differences in metabolic profiles between the two groups.

Orthogonal PLS-DA (OPLS-DA) was employed to further analyze intergroup differential metabolites and identify potential metabolic markers. Metabolites were screened using thresholds of variable importance in projection (VIP) >1 and *p* < 0.05. As illustrated in Supplementary Information Figure S4, CG and EG exhibited distinct metabolic profiles in OPLS-DA analysis. The model’s validity was assessed using R^2^Y (goodness of fit) and Q^2^ (predictive capability), where values closer to 1 indicate higher reliability. In the positive ion mode, R^2^Y and Q^2^ reached 1 and 0.987, respectively, whereas in the negative ion mode, they were 1 and 0.986, respectively, demonstrating robust predictive performance and minimal overfitting. These results confirm the high interpretability of the model for differential metabolites between groups, supporting its suitability for subsequent metabolomic investigations.

#### 3.4.2. Inter-Group Differential Analysis

**Figure 2:**
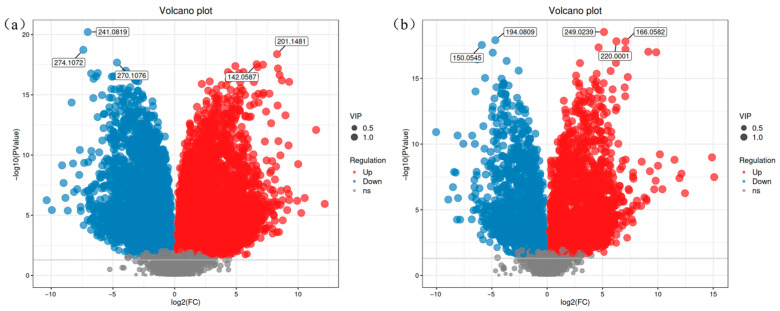
Differential metabolic volcano maps for the positive (**a**) and negative ion models (**b**). Red points represent significantly upregulated genes; blue points represent significantly downregulated genes; and the gray area represents insignificant genes.

Differential metabolites (including unidentified metabolites) across the groups were visualized using volcano plots for both ion modes (Figure 2). With screening criteria of fold change (FC) >1.5 or FC < 0.67 and *p* < 0.05, a total of 6280 metabolites were filtered in the positive ion mode, with 3538 upregulated and 2742 downregulated. Similarly, 2819 metabolites were identified in the negative ion mode, comprising 1687 upregulated and 1132 downregulated metabolites. These results highlight the significant metabolic shifts between the CG and EG.

#### 3.4.3. Cluster Analysis

**Figure 3:**
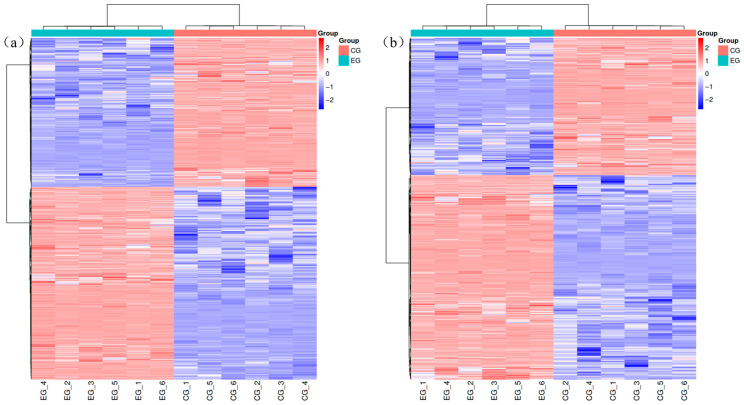
Positive (**a**) and negative (**b**) differential metabolite hierarchy clustering heatmaps. CG represents the parent strain, while EG represents the fused strain. Red denotes upregulated metabolites, blue represents downregulated metabolites, and darker hues indicate higher expression levels.

To comprehensively visualize the differences in metabolic expression between the original *V. volvacea* strain (CG group) and the fused strain SL212 (EG group), hierarchical clustering analysis was performed on differentially expressed metabolites (screened by VIP > 1 and *p* < 0.05). Metabolites with similar functions or those participating in shared metabolic pathways were grouped. This is shown in the heatmaps (Figure 3).

In the positive ion mode (Figure 3a), the upper-right red cluster signifies a higher metabolite abundance in the CG group than in the EG group, whereas the lower-left red cluster indicates an elevated abundance in the EG group. In the negative ion mode (Figure 3b), the upper-left red cluster highlights higher metabolite levels in the CG group, whereas the lower-left red cluster shows dominance in the EG group. These findings demonstrate the marked differences in the metabolic profiles between the CG and EG under identical cultivation conditions.

The Z-score (standard score) quantifies the relative abundance of metabolites by normalizing their levels across samples. As depicted in Supplementary Information Figure S5, under identical conditions, the majority of metabolites in the EG exhibited Z-scores > 0, revealing a significant increase in metabolite content compared with that in the CG.

#### 3.4.4. Correlation Analysis of Differential Metabolites

To evaluate the interrelationships among significantly differentially expressed metabolites, a correlation analysis was conducted. Positive correlations (shared trends) are depicted in red, and negative correlations (divergent trends) are indicated in blue. Each dot represents the correlation between two metabolites, with darker colors and larger dots indicating stronger associations and the absence of dots signifying no correlation. The ordinate represents differential metabolites, and the abscissa represents correlation coefficients. As illustrated in Supplementary Information Figure S6, distinct correlation patterns are observed between the CG and EG, providing a foundation for subsequent metabolite screening.

#### 3.4.5. KEGG Pathway Enrichment Analysis of Differential Metabolites

**Figure 4:**
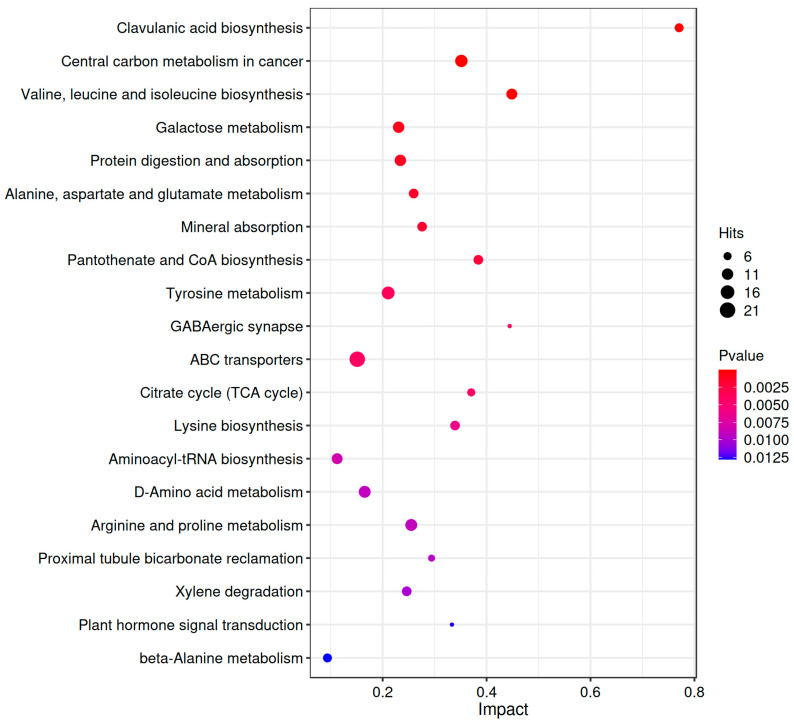
KEGG enrichment pathway. In the bubble plot, each bubble represents a metabolic pathway. The horizontal position and size of each bubble indicate the magnitude of the impact factor for that pathway in the topological analysis, with larger size corresponding to a larger impact factor. The vertical position and color of each bubble represent the *p*-value from the enrichment analysis (−log_10_ *p*-value), where a darker color indicates a smaller *p*-value and a more significant level of enrichment.

KEGG enrichment analysis was performed to identify the major metabolic and signaling pathways associated with the differential metabolites between the CG and EG. The results are displayed as a bubble plot (Figure 4), which highlights the top 20 enriched pathways. Notably, the pathways that were significantly linked to EPS synthesis included galactose metabolism, ABC transporters, and the tricarboxylic acid (TCA) cycle, suggesting their critical roles in modulating polysaccharide production.

**Figure 5:**
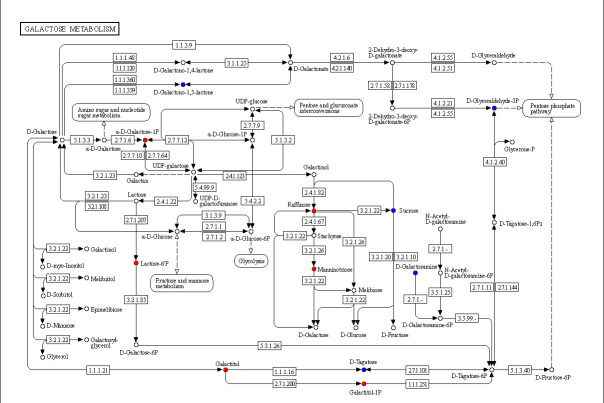
Galactose metabolic pathway. The small circles in the metabolic pathway diagram represent metabolites. Metabolites marked in red in the pathway diagram are upregulated metabolites detected in the experiment, while those in blue are downregulated metabolites.

Microbial EPS synthesis involves three key stages: nucleotide sugar precursor synthesis, repetitive unit elongation, and polymerization and export. As depicted in Figure 5, D-galactose is initially converted to α-D-galactose-1-phosphate via the action of aldose-1-epimerase (EC: 5.1.3.3) and galactokinase (EC: 2.7.1.6). This intermediate is then transformed into UDP-galactose by α-D-galactose-1-phosphate uridylyltransferase (EC: 2.7.7.10). UDP-galactose serves as a substrate for β-(1,4)-galactosyltransferase 1 (EC: 2.4.1.22), yielding UDP-glucose. Concurrently, glucose is phosphorylated by glucokinase to glucose-6-phosphate, which is subsequently isomerized to glucose-1-phosphate by α-phosphoglucomutase. Pyrophosphorylases further catalyze the synthesis of nucleotide sugars, including UDP-glucose, dTDP-glucose, UDP-glucuronic acid, UDP-galactose, and dTDP-rhamnose. Additionally, glucose-6-phosphate can be converted to mannose-6-phosphate via phosphomannomutase, followed by GDP-mannose pyrophosphorylase-mediated generation of GDP-mannose [46].

Polysaccharide synthesis occurs via four primary pathways: Wzx/Wzy-dependent, ABC transporter-dependent, synthase-dependent, and extracellular sucrase-mediated pathways [47]. In this study, metabolites that were differentially expressed between the CG and EG were predominantly enriched in the ABC transporter pathway. ABC transporters represent a major superfamily of membrane transport proteins that are ubiquitously expressed across organisms. They utilize energy from ATP hydrolysis to translocate substrates, such as sugars, peptides, lipids, and metals across biological membranes [48]. Following nucleotide sugar synthesis, repetitive units are modified by enzymes, such as methyltransferases and acetyltransferases, polymerized via protein linkages, and ultimately exported to the cell surface via ABC transporters.

Additionally, glucose-6-phosphate is isomerized to fructose-6-phosphate by phosphoglucose isomerase, which enters the TCA cycle to generate adenosine triphosphate (ATP), providing energy for polysaccharide biosynthesis. This integrated metabolic network underscores the critical roles of ABC transporters and energy metabolism in enhancing EPS production in cells of the fusion strain SL212.

#### 3.4.6. Global KEGG Pathway Variation Analysis

**Figure 6:**
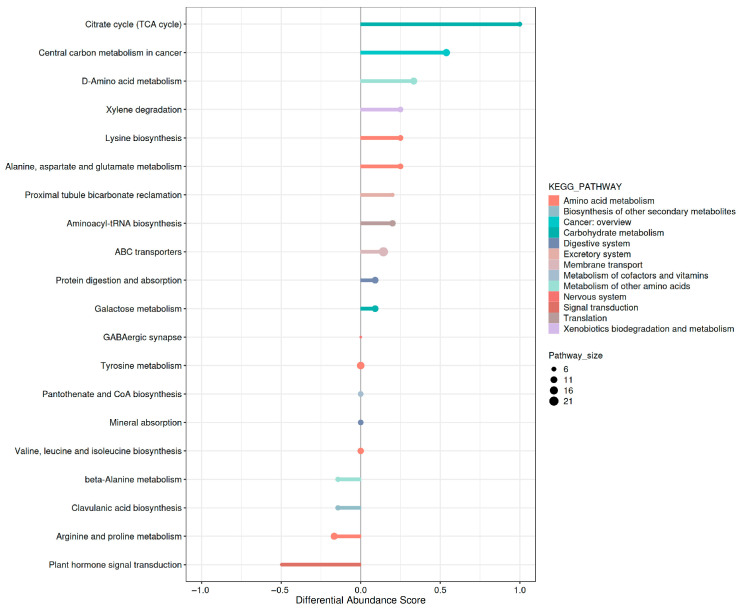
Differential abundance score chart. The y-axis lists the names of the differential pathways. The x-axis represents the Differential Abundance (DA) Score. The DA Score quantifies the overall magnitude of change for all metabolites within a metabolic pathway. A DA Score of +1 indicates up-regulated expression trends for all identified metabolites in the pathway. A DA Score of −1 indicates down-regulated expression trends for all identified metabolites in the pathway. The length of the line segment corresponds to the absolute value of the DA Score. The size of the circular marker at the end of each line segment represents the number of metabolites associated with that pathway, with larger dots indicating more metabolites.

The Differential Abundance Score is a pathway-centric metric that evaluates average collective changes in metabolites within a pathway to visualize intergroup expression disparities. The pathways were classified based on the KEGG Pathway Hierarchy, as shown in Figure 6. Each node represents a metabolic pathway, with higher scores indicating more pronounced intergroup differences. The analysis revealed 11 pathways exhibiting upregulated trends and four pathways showing downregulated trends, demonstrating that the fusant strain SL212 exhibited a more diversified metabolic profile than that of the original strain. These findings highlight robust intergroup metabolic disparities, particularly in pathways critical for polysaccharide biosynthesis and energy metabolism.

### 3.5. Transcriptomic Analysis

#### 3.5.1. Sequencing Quality Analysis and Data Quality Control

High-quality transcriptome sequencing was performed on cDNA libraries from the CG and EG using the Illumina platform. As summarized in Supplementary Information Table S6, a total of 358,552,570 filtered reads were obtained with quality scores exceeding Q20 (>99%) and Q30 (>96%), accompanied by GC content levels > 51%. These robust quality metrics confirm the reliability of the sequencing data for subsequent gene expression profiling and differential analyses.

#### 3.5.2. Analysis of Differentially Expressed Genes (DEGs)

**Figure 7:**
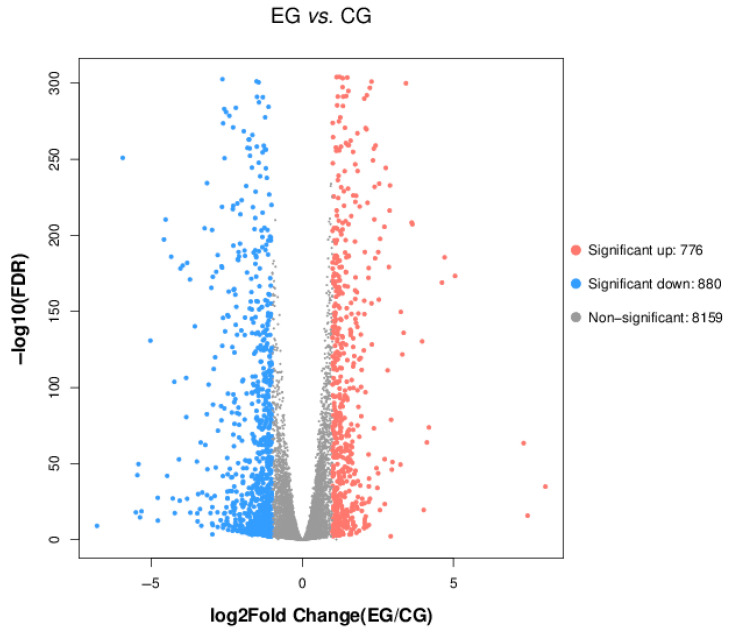
Differential metabolic volcano plot. CG represents the parent strain, while EG represents the fused strain. Red points represent significantly upregulated genes; blue points represent significantly downregulated genes; the gray area represents insignificant genes.

Volcano plot visualization revealed distinct patterns of DEGs between the parental *V. volvacea* strain and the SL212 variant (Figure 7). Stringent thresholds of |Log2Fold Change| > 1 combined with a false discovery rate (FDR) <0.05 identified 776 upregulated and 880 downregulated transcripts. The marked difference in DEG profiles between these fungal strains demonstrates their potential biosynthetic implications, particularly regarding polysaccharide metabolism-associated pathways, as evidenced by functional annotation clustering.

#### 3.5.3. Clustering Analysis

Hierarchical clustering analysis revealed distinct expression profiles of differential genes between the parental *V. volvacea* strain and the hybrid SL212 derivative. The heatmap demonstrates a dichotomous segregation pattern of 776 upregulated and 880 downregulated genes (Supplementary Information Figure S7), exhibiting pronounced intergroup divergence while maintaining strong intragroup homogeneity. Particularly noteworthy is the marked phylogenetic conservation within the biological replicates of each fungal strain, in contrast to the substantial transcriptional reprogramming events distinguishing these two genotypes.

#### 3.5.4. Enrichment Analysis of DEGs

**Figure 8:**
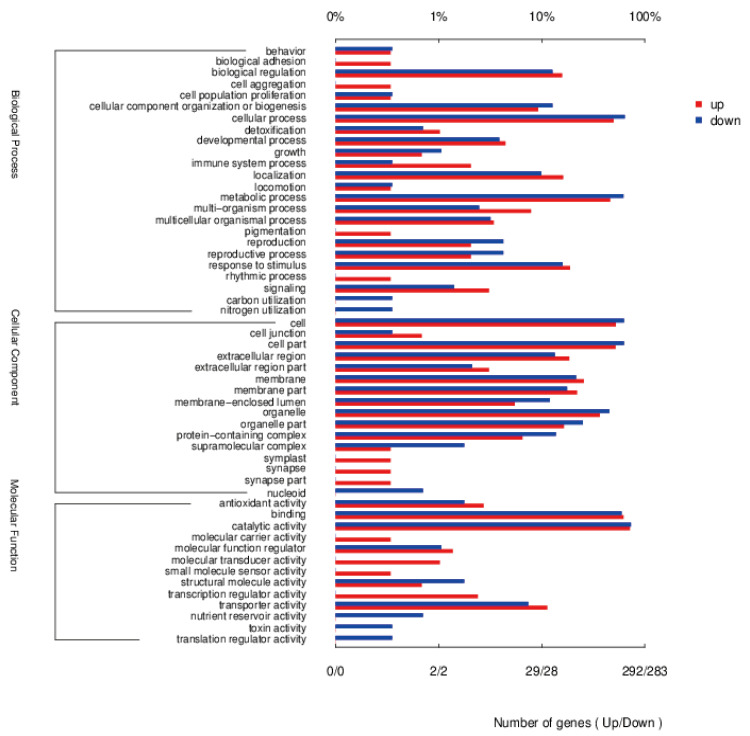
Gene ontology functional enrichment histogram. Red represents significant upregulation; blue represent significant downregulation.

Gene ontology enrichment bar charts (Figure 8) were plotted to delineate the functional categorization of DEGs, highlighting the most significantly enriched biological terms. Functional annotation clustering demonstrated a predominant association between the upregulated DEGs and biological engineering processes, followed by a statistically robust enrichment in metabolic processes, biological regulation, response to stimuli, and cellular component organization. Critical polysaccharide biosynthesis-related processes, including cellular process regulation, primary metabolism, and stress-responsive pathways, exhibited marked transcriptional upregulation in the SL212 hybrid derivative compared to that in the parental strain. This systematic reprogramming of biosynthesis-related transcriptional networks provides mechanistic insights into the enhanced EPS yield observed in the hybrid strain, which is substantiated by the coordinated activation of rate-limiting enzymes in the glycan biosynthesis and export pathways.

#### 3.5.5. KEGG Pathway Enrichment Analysis

**Figure 9:**
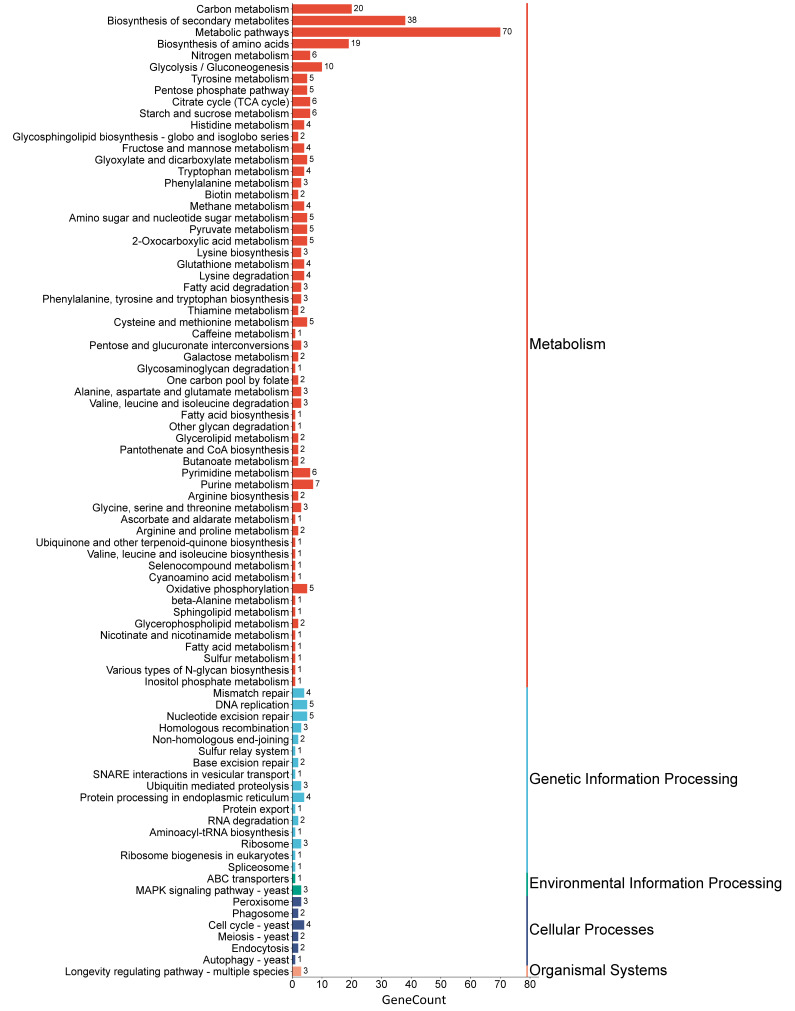
KEGG pathways classification histogram. The horizontal axis represents the number of genes. The left vertical axis represents the KEGG pathways to which the genes are annotated. The right vertical axis categorizes the KEGG pathways into five major classes, with each color representing one major class.

Functional annotation of the gene data using KEGG classification revealed that metabolic categories were predominant (Figure 9). The key pathways within this group include carbon metabolism, starch and sucrose metabolism, and the overarching metabolic pathways. The identifiers and the corresponding number of sequences for the carbohydrate metabolic pathways are presented in Supplementary Information Table S7.

#### 3.5.6. Real-Time Quantitative Polymerase Chain Reaction (RT-qPCR) Verification

**Figure 10:**
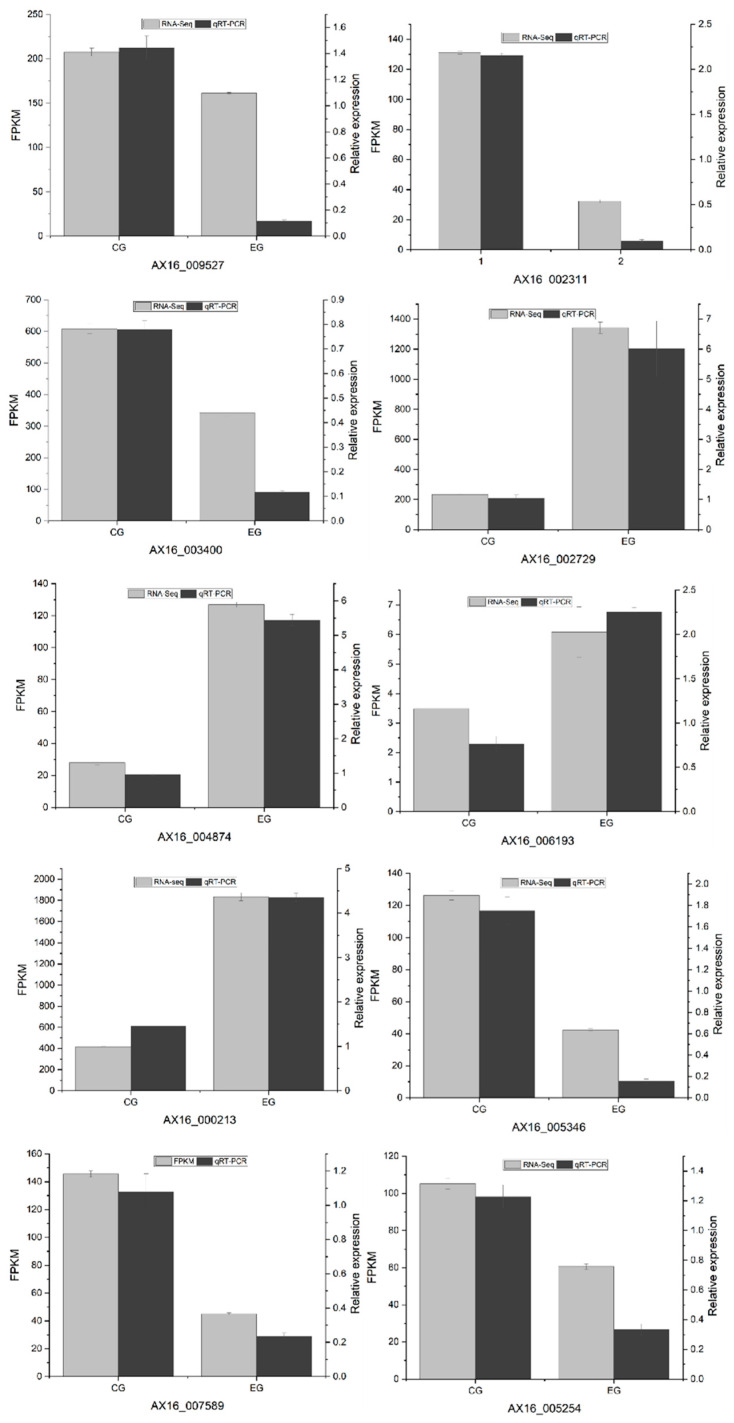
qRT-PCR validation of RNA-Seq results. CG represents the starting strain, and EG represents the fused strain. The horizontal axis indicates the gene names. The left vertical axis represents FPKM values, while the right vertical axis shows the relative expression level, illustrating both the trend of gene expression changes and relative differences.

To validate the reliability of the transcriptome sequencing data, qRT-PCR was performed on 10 randomly selected genes associated with polysaccharide synthesis within the EPS metabolic pathway, using *ACTB* as the reference gene. The expression patterns of these 10 DEGs generally aligned with the trends observed in the RNA-Seq results, thus supporting the reliability of the RNA-Seq data. The qRT-PCR verification of the RNA-Seq results is shown in Figure 10.

## 4. Discussion

*V. volvacea*, a fungal species of *Volvariella* in the family Pluteaceae, has a distinctive aromatic profile and nutritional richness [3,5]. Polysaccharides, particularly those derived from the mycelia and fruiting bodies, constitute the predominant bioactive components of this edible fungus, demonstrating significant pharmacological properties, including antioxidant efficacy, lipid metabolism regulation, and immunomodulatory functions [49]. Current research primarily focuses on enhancing cold tolerance and postharvest preservation techniques. Studies on polysaccharide yield optimization, which is a critical constraint impeding industrial-scale production, remain limited.

The biosynthesis of polysaccharides exhibits substantial strain-dependent variability [50]. To facilitate cost-effective processing and promote the industrial development of this fungal resource, strategic strain selection targeting elevated EPS production capacity represents a crucial research imperative for value-added product development.

In this study, ARTP mutagenesis was applied to *V. volvacea*, yielding nine mutant strains with polysaccharide production increased by over 20%. After three rounds of genome shuffling via protoplast fusion, a fusant strain, SL212, was obtained, exhibiting a 111.67% enhancement in EPS yield (46.85 g/L) compared to the original strain. Zhu et al. applied ARTP mutagenesis alone to *Hericium erinaceus*, achieving a 16% increase in polysaccharide yield compared to the original strain [51]. Gong et al. screened two superior strains of *Hericium erinaceus* with high mycelial polysaccharide production via ARTP mutagenesis, with their polysaccharide yields increased by 23.25% and 44.75%, respectively [52].

Metabolomic profiling revealed that the differential metabolites between SL212 and the original strain were predominantly enriched in pathways such as galactose metabolism and carbohydrate metabolism; this is consistent with the previously reported polysaccharide synthesis pathway [52,53]. These pathways not only facilitate monosaccharide synthesis but also generate ATP, providing energy for polysaccharide polymerization, thereby driving the observed EPS overproduction.

## 5. Conclusions

Through ARTP mutagenesis of *V. volvacea*, we obtained nine mutant strains exhibiting over 20% enhancement in polysaccharide yield. Following three successive rounds of genome shuffling through protoplast fusion, the fusant strain SL212 was successfully developed. Metabolomic and transcriptomics profiling revealed a significant enrichment of differential metabolites between SL212 and its parental strain in key metabolic pathways, including galactose metabolism, ABC transporter systems, and the TCA cycle. These modified pathways synergistically facilitate monosaccharide biosynthesis while generating substantial ATP reserves, thus energizing the polysaccharide polymerization process that drives EPS hyperproduction.

The findings of this study provide an efficient approach for strain improvement in the field of fungal biotechnology, offer high-quality strains and feasible methods for large-scale polysaccharide production in industrial fermentation, and supply high-activity polysaccharide raw materials for the development of functional foods. The SL212 strain can be applied in the production of functional health products, such as capsules or beverages with antioxidant and immunomodulatory effects, as well as in the pharmaceutical field as a raw material for related drugs. Future research should focus on the stability of SL212 under different fermentation conditions and the specific in vivo mechanism of action of its polysaccharides. Meanwhile, the current study has limitations, including the heavy workload of screening high-yield strains, the need for long-term monitoring of the genetic stability of the strain, and the potential oversight of other influencing factors. Nevertheless, this study lays a foundation for the industrial production of *V. volvacea* polysaccharides, points out directions for subsequent research, and will promote the in-depth application of fungal polysaccharides in various fields.

## Figures and Tables

**Table 1:** Determination results of genetic stability of SL212. Each calculation was repeated three times and is represented by SD ± average.

Passage Number	EPS Content (g/L)
1	46.02 ± 0.47
2	46.11 ± 0.58
3	46.14 ± 0.53
4	46.78 ± 0.47
5	46.05 ± 0.53

## Data Availability

The original contributions of this study are included in the article/Supplementary Material. The data presented in this study are openly available at NCBI, reference number PRJNA1303107. Further inquiries can be directed to the corresponding authors.

## Supplementary Materials

The following supporting information can be downloaded at: https://www.mdpi.com/article/10.3390/jof11080591/s1, Figure S1: Antagonistic *V. volvacea*. The number indicates the name of the strain; Figure S2: PCA scores of positive ion mode (a) and negative ion mode (b); Figure S3: PLS-DA plots of metabolites in positive (a) and negative ion mode (b); Figure S4: OPLS-DA scores in positive ion mode (a) and negative ion mode (b); Figure S5: Z-score map of differential metabolites; Figure S6: Heatmap of correlation analysis of differential metabolites; Figure S7: Differential expressions gene clustering heat map; Table S1: The primers information in this study; Table S2: Analysis of EPS contents in mutagenic strains. Each set of data was repeated three times and was represented by SD ± average; Table S3: Comparison of EPS content. Each set of data was repeated three times and was represented by SD ± average; Table S4: KEGG metabolite components annotated in the positive ion mode; Table S5: KEGG metabolite components annotated in the negative ion mode; Table S6: Evaluation and statistics of sample sequencing data; Table S7: Carbohydrate metabolic pathway number and sequence quantity information.

## Abbreviations

The following abbreviations are used in this manuscript:

KEGG	Kyoto Encyclopedia of Genes and Genomes
ARTP	Atmospheric and room-temperature plasma
EPS	Exopolysaccharide
TCA	Tricarboxylic acid cycle
ROS	Reactive oxygen species
ARE	Antioxidant response element
PEG	Polyethylene glycol
PDA	Potato Dextrose Agar
