# Supplementary material for: Breeding of High-Polysaccharide-Producing Volvariella volvacea Strains Based on Genome Shuffling Technology
Source: J Fungi (Basel). 2025 Aug 14;11(8):591. doi: 10.3390/jof11080591 (PMC12387567; doi:10.3390/jof11080591)
Supplement: Supplementary file 1 [file jof-11-00591-s001.zip › jof-3736586-supplementary.pdf]

Table S1. The primers information in this study

Gene	Forward sequence (5'-3')	Reverse sequence (5'-3')	PCR Products(bp)
ACTB	CGTTACCAACTGGGACGACA	AATAGCGACGTAGAAGGCGG	184
AX16_000213	AAAATCGATGCGCATGTCCG	GCCCTTCTACACCCTTGACC	130
AX16_009527	CGCAAACACCTGCACAATCA	ATAGCATGTCGGAGAGTGCG	96
AX16_003400	GCTTGTCGCCAGAGGTAGTT	GGATGCGCTTGACAAGAACC	138
AX16_005254	GGTAACTGGGTCTGGAACCG	CCTGGCCAAAAGAGACAGGT	146
AX16_007589	AAGAATTGGACGCTGTCGGT	TGATCGAAGCATCGCGGTAA	141
AX16_001625	AGCGTTCGCCTTGGTCAATA	CGGACCCTTGACCCTTTCTC	109
AX16_002311	TGTCCCACCTACGCCTCATA	AATCCCCGAAAGCACGATGT	189
AX16_002729	TGTCGACAACGAAGCCATCA	GGAAGTTGGATTGCGAGGGA	110
AX16_004874	AGCGTGTTAGTTCAGCGACA	TCGCTGTTCGCATTGACTCT	187
AX16_005346	CCAATTGCGCCCGAACATAG	TTGCGCATCAAGATGGGAGT	90
AX16_006193	CTCCAAGTTCACCGTCGTCA	CGAGGGTGTTGCTCATTCCT	146

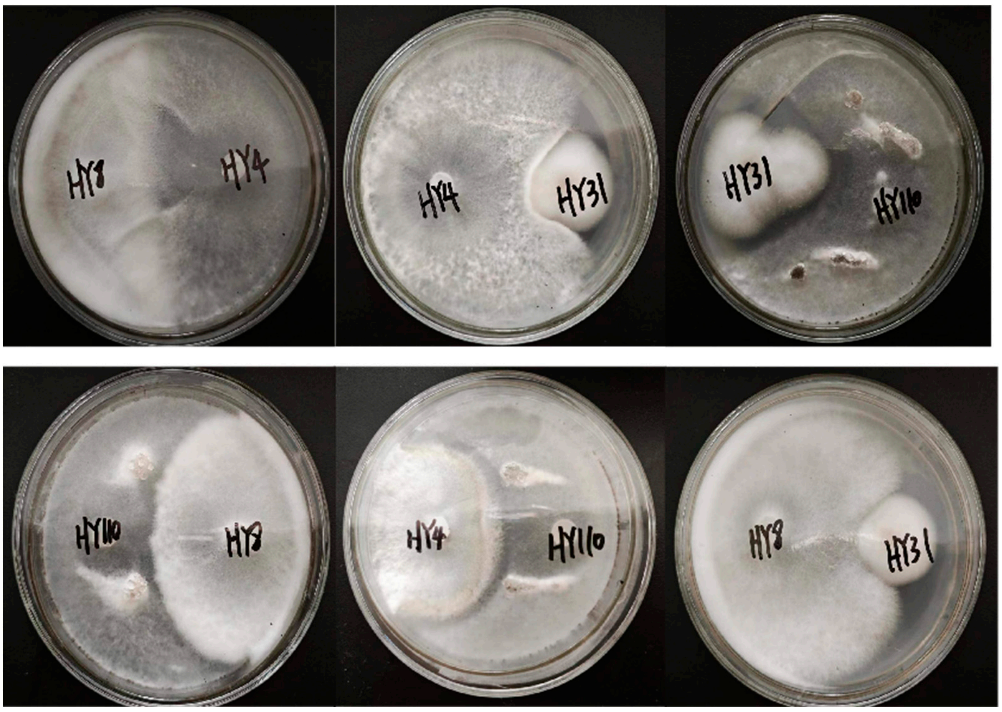

Figure S1. Antagonistic *V. volvacea*. The number indicates the name of the strain.

Table S2. Analysis of EPS contents in mutagenic strains. Each set of data was repeated three times and was represented by SD ± average.

Strain	First generation (g/L)	Second generation (g/L)	Third generation (g/L)	Fourth generation (g/L)	Fifth generation (g/L)	Increase (%)
HY110	23.24 ± 0.78	23.34±0.15	23.51±0.15	23.37±0.12	23.54 ± 0.10	6.21
HY24	35.19 ± 0.30	35.06±0.21	35.08±0.20	35.10±0.08	35.15 ± 0.20	59.40
HY31	37.16 ± 0.14	37.26±0.15	37.25±0.14	37.23±0.15	37.25 ± 0.17	68.98
HY4	35.10 ± 0.26	35.15±0.14	35.16±0.21	35.18±0.23	35.15 ± 0.35	59.53
HY15	14.67 ± 0.21	14.68±0.22	14.58±0.18	14.61±0.14	14.52 ± 0.23	-33.67
HY67	28.02 ± 0.03	28.04±0.09	28.10±0.30	28.06±0.14	28.09 ± 0.12	27.38
HY112	26.67 ± 0.10	26.51±0.25	26.54±0.26	26.54±0.30	26.60 ± 0.29	20.61
HY8	19.81 ± 0.34	19.71±0.22	19.84±0.08	19.71±0.15	19.77 ± 0.11	-10.26
HY73	27.19 ± 0.13	27.16±0.01	27.09±0.09	27.08±0.29	27.11 ± 0.08	23.13
HY106	21.74 ± 0.42	21.79±0.21	21.75±0.18	21.78±0.12	21.76 ± 0.24	-1.22
HY114	17.89 ±	17.87±0.18	17.85±0.12	17.84±0.07	17.93 ±	-18.85

	0.09				0.13	
HY123	32.09 ± 0.12	32.03±0.35	32.03±0.14	32.03±0.11	32.07 ± 0.28	45.47
HY103	32.74 ± 0.06	32.72±0.05	32.77±0.19	32.80±0.11	32.82 ± 0.22	48.74
HY105	21.18 ± 0.28	21.14±0.24	21.22±0.40	21.22±0.28	21.20 ± 0.24	-3.81
HY115	37.89 ± 0.09	37.81±0.09	37.86±0.11	37.67±0.14	37.70 ± 0.32	71.52
HY36	9.65±0.19	9.68±0.32	9.62±0.12	9.63±0.25	9.74±0.23	-56.14
HY17	14.45 ± 0.42	14.45±0.14	14.47±0.21	14.40±0.16	14.40 ± 0.13	-34.48
HY129	21.97 ± 0.04	22.01±0.20	21.96±0.09	21.90±0.08	21.92 ± 0.44	-0.36
HY132	19.71 ± 0.26	19.74±0.29	19.77±0.10	19.70±0.15	19.71 ± 0.27	-10.71

Table S3. Comparison of EPS content. Each set of data was repeated three times and was represented by SD ± average.

Strain	EPS content (g/L)	Increase (%)
V. volvacea	22.13±0.09	-
SL159	24.22±0.76	9.46
SL183	30.73±0.20	38.87
SL188	38.19±0.56	72.56
SL212	46.85±0.45	111.67

SL217	40.33±0.73	82.20
SL225	33.82±1.13	52.82
SL231	32.02±0.39	44.69
SL233	32.63±0.54	47.41
SL241	44.27±0.44	100.02

Table S4. KEGG metabolite components annotated in the positive ion mode

Number	Metabolite	Formula	KEGG Compound ID	M/Z	ppm
1	SCHEMBL534447	$C_5H_{11}NO$	C03982	102.0551	1.444
2	Benzimidazole	$C_7H_6N_2$	C02009	102.0341	2.714581694
3	2-Methylcholine	$C_6H_{16}NO^+$	C02224	102.1279	1.772
4	4-Vinylphenol	C_8H_8O	C05627	103.0546	3.656
5	(Z)-3-aminoperacrylic acid	$C_3H_5NO_3$	C20249	104.0347	4.629031467
6	Indole-5,6-quinone	$C_8H_5NO_2$	C05579	104.0495	0.255454375
7	(S)-2-Methylbutanal	$C_5H_{10}O$	C02223	104.1073	3.012
8	γ-Aminobutyric acid	$C_4H_9NO_2$	C00334	104.0709	2.865
9	Phenylethylamine	$C_8H_{11}N$	C05332	105.0702	3.122
10	Hydroxyhydroquinone	$C_6H_6O_3$	C02814	109.0283	0.954065137
11	p-Cresol	C_7H_8O	C01468	109.0651	2.859
12	o-Cresol	C_7H_8O	C01542	109.0652	2.859
13	Arsenite	AsH_3O_3	C06697	109.0224	18.927
14	Phenylhydrazine	$C_6H_8N_2$	C02304	109.0762	1.639
15	aminophenol	C_6H_7NO	C01987	110.0609	6.926897678

16	2-Aminoacrylic acid	$C_3H_5NO_2$	C02218	110.0198	13.15854146
17	Hydroxylaminobenzene	C_6H_7NO	C02720	110.0599	1.250410913
18	Patulin	$C_7H_6O_4$	C16748	111.0441	0.414068825
19	2,3,4,5-Tetrahydro-2-pyridinecarboxylic acid	$C_6H_9NO_2$	C00450	111.0441	0.424875342
20	Acrylic acid	$C_3H_4O_2$	C00511	111.1172	15.117
21	Aniline	C_6H_7N	C00292	111.0919	2.055774543
22	Capillene	$C_{12}H_{10}$	C16927	111.01	17.191
23	Barbituric acid	$C_4H_4N_2O_3$	C00813	111.02	9.883
24	Imidazole-4-acetaldehyde	$C_5H_6N_2O$	C05130	111.0557	3.718673603
25	2,6-Dihydroxypyridine	$C_5H_5NO_2$	C03056	112.0395	1.754558883
26	Phenol	C_6H_6O	C00146	112.0757	0.107
27	3-Chlorophenol	C_6H_5ClO	C14270	111.9847	9.658282783
28	3,4-Pyridinediol	$C_5H_5NO_2$	C02932	112.0395	1.754558883
29	Vigabatrin	$C_6H_{11}NO_2$	C07500	113.0597	0.018
30	2-Furancarboxaldehyde	$C_5H_4O_2$	C14279	114.055	0.4048915
31	Aminocaproic acid	$C_6H_{13}NO_2$	C02378	114.092	5.827
32	Paramethadione	$C_7H_{11}NO_3$	C07411	114.0914	0.561
33	L-Prolinamide	$C_5H_{10}N_2O$	C19781	115.0391	1.139
34	Tulipalin B	$C_5H_6O_3$	C21187	115.0396	5.485
35	3-Hydroxy-L-proline	$C_5H_9NO_3$	C05147	115.0754	0.424
36	L-Leucine	$C_6H_{13}NO_2$	C00123	115.0756	2.161882276
37	(S)-2-Acetolactate	$C_5H_8O_4$	C06010	115.039	0.276253271
38	Isopropylmaleic acid	$C_7H_{10}O_4$	C02631	115.0756	2.151454348
39	2,4-Diaminopentanoate	$C_5H_{12}N_2O_2$	C03943	116.0713	0.005
40	alpha-Methylene-gamma-butyrolactone	$C_5H_6O_2$	C20578	116.0707	0.836
41	L-2-Amino-4-methylenepentanedioic acid	$C_6H_9NO_4$	C00651	116.0713	0.846

42	Cyclohexanone	$C_6H_{10}O$	C00414	116.0706	0.026
43	4-methylthiobutanaldoxime	$C_5H_{11}NOS$	C17241	116.0527	1.232372017
44	Glycylglycine	$C_4H_8N_2O_3$	C02037	116.0354	10.193
45	L-Proline	$C_5H_9NO_2$	C00148	116.0707	0.846
46	5-Hydroxybenzimidazole	$C_7H_6N_2O$	C21764	117.0701	1.944
47	alpha-Ketoisovaleric acid	$C_5H_8O_3$	C00141	117.0545	1.009957755
48	Cyclohexanol	$C_6H_{12}O$	C00854	118.0858	3.83
49	Succinic anhydride	$C_4H_4O_3$	C19524	118.0858	3.83
50	Isovaline	$C_5H_{11}NO_2$	C03571	118.0857	4.666
51	Glutaral	$C_5H_8O_2$	C12518	118.0857	4.676
52	L-Valine	$C_5H_{11}NO_2$	C00183	118.0859	2.973
53	Carnitine	$C_7H_{15}NO_3$	C00318	118.1203	19.763
54	Benzeneacetonitrile	C_8H_7N	C16074	118.0654	2.349375007
55	2-Phenylacetamide	C_8H_9NO	C02505	118.0654	2.356
56	(3Z,6Z)-3,6-Nonadienal	$C_9H_{14}O$	C16323	122.0714	9.932
57	2,4-DMA	$C_8H_{11}N$	C11003	122.0967	1.466
58	2-Phenylethanol	$C_8H_{10}O$	C05853	123.0801	0.508446911
59	4-Hydroxybenzaldehyde	$C_7H_6O_2$	C00633	123.0442	1.186402122
60	3-METHYLBENZYL ALCOHOL	$C_8H_{10}O$	C07216	123.0807	2.133396219
61	Carbamic acid	CH_3NO_2	C01563	123.0406	4.616199043
62	L-Serine	$C_3H_7NO_3$	C00065	123.0755	7.447624426
63	4-Amino-5-hydroxymethyl-2-methylpyrimidine	$C_6H_9N_3O$	C01279	123.0793	1.667
64	Niacinamide	$C_6H_6N_2O$	C00153	123.0554	0.918
65	Isonicotinamide	$C_6H_6N_2O$	C02421	123.0559	0.707
66	p-Xylene	C_8H_{10}	C06756	124.1123	1.841718347
67	Benzaldehyde	C_7H_6O	C00261	124.076	2.514425836
68	Pyrazinamide	$C_5H_5N_3O$	C01956	124.0388	4.059
69	L-Histidinol	$C_6H_{11}N_3O$	C00860	124.0864	4.182731548

70	2,5-Furandicarbaldehyde	$C_6H_4O_3$	C20899	125.0233	0.15853125
71	Nitrosobenzene	C_6H_5NO	C06876	125.0709	0.299191099
72	L-Hypoglycin A	$C_7H_{11}NO_2$	C08287	125.0236	2.251
73	4-Methyl-5-nitrocatechol	$C_7H_7NO_4$	C18315	126.0541	6.763921998
74	Tartronate semialdehyde	$C_3H_4O_4$	C01146	127.0017	11.97542946
75	Pyruvic acid	$C_3H_4O_3$	C00022	127.1125	10.18641046
76	Glyceraldehyde 3-phosphate	$C_3H_7O_6P$	C00118	127.0156	1.0107412
77	3,4-Dihydroxyphenylglycol	$C_8H_{10}O_4$	C05576	127.0766	9.030622499
78	Malonate	$C_3H_4O_4$	C00383	127.0016	11.18804724
79	Thymine	$C_5H_6N_2O_2$	C00178	127.0501	0.802991103
80	1,3-Benzenediol	$C_6H_6O_2$	C01751	128.07	3.927693505
81	5-Amino-4-imidazole carboxylate	$C_4H_5N_3O_2$	C05516	128.0439	12.11631401
82	Methylenecyclopropylglycine	$C_6H_9NO_2$	C08292	128.0701	3.918
83	5-Methyl-2-furancarboxaldehyde	$C_6H_6O_2$	C11115	128.0704	1.585
84	Tetrahydrodipicolinate	$C_7H_9NO_4$	C03972	128.0701	3.918323637
85	FA 6_1;O2	$C_6H_{10}O_4$	C14463	130.0859	2.689
86	Benzylamine	C_7H_9N	C15562	130.0652	19.098
87	N4-Acetylaminobutanal	$C_6H_{11}NO_2$	C05936	130.0859	1.913956483
88	Chloroacetyl chloride	$C_2H_2Cl_2O$	C14859	130.0486	9.756
89	Uracil	$C_4H_4N_2O_2$	C00106	130.0873	8.055
90	Coumarin	$C_9H_6O_2$	C05851	130.0169	4.653395828
91	N-Acetyl-L-glutamate 5-semialdehyde	$C_7H_{11}NO_4$	C01250	130.1201	19.477
92	Isoglutamine	$C_5H_{10}N_2O_3$	C16673	130.0974	10.96
93	L-Glutamine	$C_5H_{10}N_2O_3$	C00064	130.0495	2.818

94	3-Thiomorpholinecarboxylic acid	$C_5H_9NO_2S$	C03901	130.0492	5.127
95	O-Acetylserine	$C_5H_9NO_4$	C00979	130.0489	7.434281259
96	Menadiol	$C_{11}H_{10}O_2$	C07126	131.0857	1.354685523
97	1-Pyrroline-5-carboxylic acid	$C_5H_7NO_2$	C04322	131.0815	0.012
98	(R)-demethyl-4-deoxygadusol	$C_7H_{10}O_5$	C21281	131.0703	0.248568135
99	Anisole	C_7H_8O	C01403	131.0491	18.066
100	L-Methionine	$C_5H_{11}NO_2S$	C00073	132.0478	0.317914422
101	indole-3-glycol aldehyde	$C_{10}H_9NO_2$	C03230	132.0809	0.970465071
102	Indoleacetic acid	$C_{10}H_9NO_2$	C00954	132.0809	0.970465071
103	Beta-Leucine	$C_6H_{13}NO_2$	C02486	132.1002	12.871
104	Mevalonic acid	$C_6H_{12}O_4$	C00418	132.0551	6.189688244
105	2-Heptanone	$C_7H_{14}O$	C08380	132.1009	7.581
106	(R)-2,3-Dihydroxy-3-methylvalerate	$C_6H_{12}O_4$	C06007	132.0557	10.73319743
107	Propiobetaine	$C_6H_{13}NO_2$	C08263	132.1003	12.11367499
108	(R)-Carvone	$C_{10}H_{14}O$	C01767	133.1026	10.73743037
109	Ureidosuccinic acid	$C_5H_8N_2O_5$	C00438	133.0589	3.503825709
110	Dimethyl sulphone	$C_2H_6O_2S$	C11142	133.0974	3.764065659
111	3-Hydroxy-2-oxoindole	$C_8H_7NO_2$	C11130	133.0317	0.724505651
112	Asparagine	$C_4H_8N_2O_3$	C00152	133.0603	3.504
113	D-Ribose	$C_5H_{10}O_5$	C00121	133.0496	0.508
114	Guanine	$C_5H_5N_5O$	C00242	134.045	8.351076877
115	4-Hydroxybenzeneacetonitrile	C_8H_7NO	C03766	134.0586	10.72381854
116	(R)-mandelic Acid	$C_8H_8O_3$	C01983	135.0804	0.271
117	Salicylhydroxamic acid	$C_7H_7NO_3$	C11343	136.0762	3.777
118	Thiobenzamide S-oxide	C_7H_7NOS	C16283	136.0216	0.407
119	(R)-Amphetamine	$C_9H_{13}N$	C07514	136.062	1.695
120	Dextroamphetamine	$C_9H_{13}N$	C07884	136.0757	0.097
121	Adenine	$C_5H_5N_5$	C00147	136.0622	1.695
122	Piperitone	$C_{10}H_{16}O$	C09885	136.062	18.025

123	Dopamine	$C_8H_{11}NO_2$	C03758	137.0593	2.933
124	4-Aminobenzoic acid	$C_7H_7NO_2$	C00568	138.0544	4.003
125	2-Aminobenzoic acid	$C_7H_7NO_2$	C00108	138.0544	4.003
126	3-Methylbenzaldehyde	C_8H_8O	C07209	138.0919	4.075
127	2-Hydroxyphenethylamine	$C_8H_{11}NO$	C02735	138.0911	1.709
128	o-Ethyltoluene	C_9H_{12}	C14572	138.0917	2.627
129	Mesitylene	C_9H_{12}	C14508	138.1278	0.575
130	1-Methylnicotinamide	$[C_7H_9N_2O]+$	C02918	138.0549	0.381
131	4-Methylbenzaldehyde	C_8H_8O	C06758	138.0914	0.455
132	3-Methoxy-4-hydroxyphenylglycolaldehyde	$C_9H_{10}O_4$	C05583	139.0755	1.06114952
133	3-Ethyl-1,2-benzenediol	$C_8H_{10}O_2$	C06728	139.0753	0.377
134	1-Chloro-2-nitrobenzene	$C_6H_4ClNO_2$	C14407	139.9879	13.33558186
135	4-Nitrophenol	$C_6H_5NO_3$	C00870	140.0276	0.13154001
136	N-Ethylammelide	$C_5H_8N_4O_2$	C06558	140.0705	8.858252808
137	3-Indoleacetonitrile	$C_{10}H_8N_2$	C02938	140.0706	19.142
138	3-Hydroxypicolinic acid	$C_6H_5NO_3$	C18620	140.0342	0.132
139	5-Chloro-3-methylcatechol	$C_7H_7ClO_2$	C03591	141.0103	0.945887641
140	3-Methylcrotonylglycine	$C_7H_{11}NO_3$	C20828	141.0543	2.247
141	3-Buten-2-one	C_4H_6O	C20701	141.091	0.01148266
142	Methylmalonic acid	$C_4H_6O_4$	C02170	141.0149	6.583
143	4-Methylcatechol	$C_7H_8O_2$	C06730	142.0868	3.855
144	3-methylcatechol	$C_7H_8O_2$	C02923	142.0869	3.855
145	Indoleacetaldehyde	$C_{10}H_9NO$	C00637	143.0722	5.219882688
146	Muconolactone	$C_6H_6O_4$	C04105	143.0337	1.286550652

147	betaine aldehyde hydrate	$C_5H_{14}NO_2+$	C07345	143.0904	8.869219039
148	4-carboxymuconolactone	$C_7H_6O_6$	C01278	143.0338	0.587414303
149	4-carboxy-2-hydroxymuconate semialdehyde hemiacetal	$C_7H_6O_6$	C05375	143.0337	1.286550652
150	2-Methyl-4-quinolinol	$C_{10}H_9NO$	C21873	143.0491	0.264385445
151	2-(dihydroxymethyl)-5-formylfuran	$C_6H_6O_4$	C20900	143.0338	0.587414303
152	3-Oxoadipate enol-lactone	$C_6H_6O_4$	C03586	143.0337	1.286550652
153	D-proline betaine	$C_7H_{13}NO_2$	C21514	144.1018	0.69548125
154	Tryptophol	$C_{10}H_{11}NO$	C00955	144.0808	0.201136446
155	N-Formyl-L-aspartate	$C_5H_7NO_5$	C01044	144.0643	8.441
156	Hygroline	$C_8H_{17}NO$	C10152	144.0818	7.136
157	Pseudoconhydrine	$C_8H_{17}NO$	C10168	144.0641	7.058
158	Sulcatone	$C_8H_{14}O$	C07287	144.1025	4.154
159	Stachydrine	$C_7H_{13}NO_2$	C10172	144.1016	1.386377437
160	5-(2-Hydroxyethyl)-4-methylthiazole	C_6H_9NOS	C04294	144.0472	3.879
161	2-Formylglutarate	$C_6H_8O_5$	C16159	144.0808	18.798
162	D-Threitol	$C_4H_{10}O_4$	C16884	145.048	6.02076485
163	Malondialdehyde	$C_3H_4O_2$	C19440	145.048	10.57
164	Cyanurodiamide	$C_3H_5N_5O$	C08733	145.0841	5.972948793
165	2-Methylpropanal	C_4H_8O	C22919	145.1223	0
166	Valproic acid	$C_8H_{16}O_2$	C07185	145.0506	6.665
167	Butanal	C_4H_8O	C01412	145.0506	6.665
168	3,4-Dichloroaniline	$C_6H_5Cl_2N$	C02791	144.9826	12.68717832
169	6-methylthiohexanaldoxime	$C_7H_{15}NOS$	C17246	145.0608	6.497454661
170	L-Rhamno-1,4-lactone	$C_6H_{10}O_5$	C02991	145.0505	6.670641604
171	Pyruvaldehyde	$C_3H_4O_2$	C00546	145.0485	7.12327325
172	Ketopantolactone	$C_6H_8O_3$	C01125	146.0815	2.278042737

173	FA 6_2;O	$C_6H_8O_3$	C06761	146.082	2.278
174	Spermidine	$C_7H_{19}N_3$	C00315	146.1651	0.450313378
175	7-hydroxylysine	$C_6H_{14}N_2O_3$	C01028	146.0812	0.241
176	4-Guanidinobutanoic acid	$C_5H_{11}N_3O_2$	C01035	146.0923	0.677791369
177	ACMC-20my24	$C_5H_6O_5$	C06316	147.0306	12.24901415
178	L-Lysine	$C_6H_{14}N_2O_2$	C00047	147.1127	0.672
179	Pipecolic acid	$C_6H_{11}NO_2$	C00408	147.0766	1.247
180	(S)-2-Aceto-2-hydroxybutanoic acid	$C_6H_{10}O_4$	C06006	147.0651	0.560432761
181	L-trihomomethionine	$C_8H_{17}NO_2S$	C17221	148.1162	5.126919945
182	L-Glutamic acid	$C_5H_9NO_4$	C00025	148.0612	5.188
183	Ephedrine	$C_{10}H_{15}NO$	C01575	149.0961	0.097788608
184	Phthalic acid	$C_8H_6O_4$	C01606	149.024	4.57
185	Ethionamide	$C_8H_{10}N_2S$	C07665	150.0764	6.779
186	Racemethionine	$C_5H_{11}NO_2S$	C01733	150.0582	0.82
187	p-Tolyl acetate	$C_9H_{10}O_2$	C01963	151.0761	4.948
188	Cuminyl alcohol	$C_{10}H_{14}O$	C06576	151.1113	2.884
189	Xylose	$C_5H_{10}O_5$	C00216	151.0757	2.301
190	(S)-Carvone	$C_{10}H_{14}O$	C11383	151.1113	2.884
191	2-Hydroxyadenine	$C_5H_5N_5O$	C74731	152.0702	0.67
192	trans-Cinnamyl alcohol	$C_9H_{10}O$	C02394	152.0701	3.308
193	Acetaminophen	$C_8H_9NO_2$	C06804	152.0712	3.934
194	P-Hydroxyphenylethanolamine	$C_8H_{11}NO_2$	C04227	154.0865	1.616
195	M-toluic Acid	$C_8H_8O_2$	C07211	154.0863	2.935
196	Benzyl formate	$C_8H_8O_2$	C05613	154.0867	2.286
197	3-Hydroxyanthranilic acid	$C_7H_7NO_3$	C00632	154.0499	0.210190984
198	L-Glutamic gamma-semialdehyde	$C_5H_9NO_3$	C01165	154.0606	12.371
199	4-Nitrotoluene	$C_7H_7NO_2$	C14394	155.0812	0.010446765
200	Trigonelline (N'-methylnicotinate)	$C_7H_7NO_2$	C01004	155.0809	3.879395206
201	Glutaric acid	$C_5H_8O_4$	C00489	155.0338	14.981

202	Fumaric acid	$C_4H_4O_4$	C00122	155.1066	13.60238765
203	L-Ornithine	$C_5H_{12}N_2O_2$	C00077	155.0816	16.16
204	3-ureidopropionate	$C_4H_8N_2O_3$	C02642	155.0451	15.412
205	4-(hydroxymethyl)-2-methylphenol	$C_8H_{10}O_2$	C73536	156.102	0.009097263
206	Tyrosol	$C_8H_{10}O_2$	C06044	156.1019	0.631509526
207	(2E,4E,6E)-7-hydroxy-4-methylhepta-2,4,6-trienal	$C_8H_{10}O_2$	C20694	156.102	0.631509526
208	4-Hydroxybenzoic acid	$C_7H_6O_3$	C00156	156.0656	0.525291288
209	L-Histidine	$C_6H_9N_3O_2$	C00135	156.0766	0.96
210	Deisopropylatrazine	$C_5H_8ClN_5$	C06556	157.0502	7.362105238
211	1,8-Dihydroxy-3-methylnaphthalene	$C_{11}H_{10}O_2$	C12344	157.0648	0.080093694
212	Valienone	$C_7H_{10}O_5$	C17696	157.0488	4.663646586
213	2,4,5-Trihydroxytoluene	$C_7H_8O_3$	C18317	158.0804	5.485946699
214	Phenylmethylsulfonyl fluoride	$C_7H_7FO_2S$	C06747	158.0028	2.633
215	Gentisyl alcohol	$C_7H_8O_3$	C10792	158.0803	5.485946699
216	L-Homoserine	$C_4H_9NO_3$	C00263	158.154	12.59418099
217	Citrulline	$C_6H_{13}N_3O_3$	C00327	158.0922	1.254
218	L-Threonine	$C_4H_9NO_3$	C00188	158.154	12.594
219	L-Theanine	$C_7H_{14}N_2O_3$	C01047	158.0802	6.103
220	4-Oxalomesaconate	$C_7H_6O_7$	C04434	159.0308	12.58234191
221	2,5-Dichloro-1,4-benzoquinone	$C_6H_2Cl_2O_2$	C21104	158.9861	11.024
222	cis-3-Hexenyl acetate	$C_8H_{14}O_2$	C19757	160.1332	0.001
223	N-formylmethionine	$C_6H_{11}NO_3S$	C03145	160.0427	0.169
224	Cysteinylglycine	$C_5H_{10}N_2O_3S$	C01419	161.0359	12.55881515
225	Phenacetin	$C_{10}H_{13}NO_2$	C07591	162.056	6.468
226	4-hydroxy-2,2-bipyrrole-5-methanol	$C_9H_{10}N_2O_2$	C21568	162.0757	18.88265854
227	Aesculetin	$C_9H_6O_4$	C09263	162.0303	5.209026336
228	Nicotine	$C_{10}H_{14}N_2$	C00745	163.1229	0.422
229	Anabasine	$C_{10}H_{14}N_2$	C06180	163.1229	0.422

230	Coniferyl alcohol	$C_{10}H_{12}O_3$	C00590	163.0758	2.749518322
231	Ethyl trans-p-methoxycinnamate	$C_{12}H_{14}O_3$	C10476	163.1118	0.393
232	D-Mannose	$C_6H_{12}O_6$	C00936	163.0612	6.767
233	4-Oxo-1-(3-pyridyl)-1-butanone	$C_9H_9NO_2$	C19567	164.07	3.668069117
234	Theophylline	$C_7H_8N_4O_2$	C07130	164.0646	6.227
235	Eugenol	$C_{10}H_{12}O_2$	C10453	165.0911	0.596
236	Fomepizole	$C_4H_6N_2$	C07837	165.1132	1.621
237	Benzoylacetate	$C_9H_8O_3$	C07114	165.0513	6.169
238	Phenylpyruvate	$C_9H_8O_3$	C00166	165.0557	6.554
239	(R)-2-Hydroxy-2H-1,4-benzoxazin-3(4H)-one	$C_8H_7NO_3$	C15769	166.0498	0.407227832
240	1-Methyluric acid	$C_6H_6N_4O_3$	C16359	166.0494	5.269395132
241	D-Talitol	$C_6H_{14}O_6$	C21524	166.0581	10.00746185
242	4-Pyridoxolactone	$C_8H_7NO_3$	C00971	166.0499	0.195001021
243	Normetanephrine	$C_9H_{13}NO_3$	C05589	166.0866	2.106009154
244	Hydroxylated lecithin	$C_4H_8N_2O_4$	C03124	166.0855	19.688
245	Dihydroconiferyl alcohol	$C_{10}H_{14}O_3$	C10448	166.0977	6.778
246	2,4-DINITROTOLUENE	$C_7H_6N_2O_4$	C91637	166.0349	14.40673075
247	9-Hydroxyfluorene	$C_{13}H_{10}O$	C06711	166.0526	7.759108258
248	Choline sulfate	$C_5H_{13}NO_4S$	C00919	166.0523	5.644125977
249	L-Arabonolactone	$C_5H_8O_5$	C01114	166.0724	8.4467973
250	3,4-Dihydro-2H-1-benzopyran-2-one	$C_9H_8O_2$	C02274	166.0863	0.288
251	4-Ethyl-1,2-dimethoxybenzene	$C_{10}H_{14}O_2$	C75755	167.1066	0.304
252	3-Methyl-1-(2,4,6-trihydroxyphenyl)-1-butanone	$C_{11}H_{14}O_4$	C07350	167.1064	1.500960466
253	Vanylglycol	$C_9H_{12}O_4$	C05594	167.0711	4.988175094
254	3-(3-hydroxyphenyl)propionate	$C_9H_{10}O_3$	C11457	167.0682	12.375
255	3-Methylxanthine	$C_6H_6N_4O_2$	C16357	167.0565	0.898
256	FAMP	$C_7H_{10}N_4O$	C19872	167.0927	5.586271139

257	(2E,4E,6E)-4-methylocta-2,4,6-trienedial	$C_9H_{10}O_2$	C20697	168.1014	2.982843094
258	3,4-Dihydroxymandelaldehyde	$C_8H_8O_4$	C05577	169.0497	0.986573179
259	(3S)-3,6-Diaminohexanoate	$C_6H_{14}N_2O_2$	C01142	169.0963	9.799
260	Homogentisic acid	$C_8H_8O_4$	C00544	169.0486	5.52
261	(Z)-[(4-hydroxyphenyl)acetaldehyde oxime]	$C_8H_9NO_2$	C04353	169.0961	0.301
262	(3S,5S)-3,5-Diaminohexanoate	$C_6H_{14}N_2O_2$	C01186	169.0961	8.024
263	6-oxocineole	$C_{10}H_{16}O_2$	C00848	169.1226	1.773742244
264	(1R,4S)-1-hydroxylimonen-2-one	$C_{10}H_{16}O_2$	C19083	169.1225	1.774
265	4-isopropenyl-7-methyloxepan-2-one	$C_{10}H_{16}O_2$	C11414	169.1219	1.773986235
266	4-Hydroxyphenylacetaldoxime	$C_8H_9NO_2$	C04350	169.0963	0.29083805
267	Acetylcholine	$[C_7H_{16}NO_2]+$	C01996	169.0457	8.44
268	Vanillin	$C_8H_8O_3$	C00755	170.0817	2.747049489
269	Demanyl phosphate	$C_4H_{12}NO_4P$	C13482	170.06	13.70916088
270	4-hydroxyphenylacetate	$C_8H_8O_3$	C00642	170.0809	1.571135265
271	Psoralen	$C_{11}H_6O_3$	C09305	170.0356	3.699343549
272	Iminoaspartic acid	$C_4H_5NO_4$	C05840	170.1182	8.279067731
273	Methyl 2-hydroxybenzoate	$C_8H_8O_3$	C12305	170.0805	3.922966478
274	D-Glycerate 3-phosphate	$C_3H_7O_7P$	C00197	169.9975	0.137530846
275	Isopropyl catechol	$C_9H_{12}O_2$	C01014	170.1181	3.229402985
276	biphenol	$C_{12}H_{10}O_2$	C03209	170.0467	12.36966139
277	Homo-L-arginine	$C_7H_{16}N_4O_2$	C01924	171.1129	0.596

278	6-Chloro-N-(1-methylethyl)-1,3,5-triazine-2,4-diamine	$C_6H_{10}ClN_5$	C06559	171.066	5.877381245
279	Deoxyribose 5-phosphate	$C_5H_{11}O_7P$	C00673	171.0437	11.78049762
280	N8-Acetylspermidine	$C_9H_{21}N_3O$	C01029	171.1491	0.473
281	Tabtoxinine-delta-lactam	$C_7H_{12}N_2O_4$	C20920	171.0772	0.499074391
282	N2-acetyllysine	$C_8H_{16}N_2O_3$	C12989	171.1129	0.596
283	Citronellic acid	$C_{10}H_{18}O_2$	C16462	171.138	0.296719022
284	SCHEMBL5940872	$C_8H_{15}NO_4$	C06326	172.0967	0.665
285	L-Aspartic acid	$C_4H_7NO_4$	C00049	172.1343	5.563214885
286	Indolin-2-one	C_8H_7NO	C12312	172.1524	10.96342485
287	(-)-Isodihydrocarveol	$C_{10}H_{18}O$	C11411	172.1695	0.483361455
288	8-methylthiooctanaldoxime	$C_9H_{19}NOS$	C17251	172.1145	5.46043535
289	(S)-Ureidoglycolic acid	$C_3H_6N_2O_4$	C00603	173.1303	1.195747365
290	gamma-Butyrolactone	$C_4H_6O_2$	C01770	173.079	10.582
291	5,6-dihydroxy-3-methylquinolin-2(1H)-one	$C_{10}H_9NO_3$	C06725	174.0553	2.000398149
292	Indole-3-acetamide	$C_{10}H_{10}N_2O$	C02693	175.0868	1.226
293	8-methylnaphthalene-1,2-diol	$C_{11}H_{10}O_2$	C14084	175.0755	0.843
294	L-Arginine	$C_6H_{14}N_4O_2$	C00062	175.119	0.304
295	Isobutanal oxime	C_4H_9NO	C03219	175.1441	0.016
296	Geranial	$C_{10}H_{16}O$	C01499	175.1078	9.875642748
297	Isoferulic acid	$C_{10}H_{10}O_4$	C10470	177.0553	3.855
298	1-Hydroxyphenanthrene	$C_{14}H_{10}O$	C11432	178.05	15.797
299	Methyleugenol	$C_{11}H_{14}O_2$	C10454	179.1063	1.958725628
300	3,7-Dimethyluric acid	$C_7H_8N_4O_3$	C16360	180.0651	5.141362207
301	4-Hydroxycoumarin	$C_9H_6O_3$	C20414	180.0652	1.766138599

302	3,5-Dimethylphenyl methylcarbamate	$C_{10}H_{13}NO_2$	C18771	180.1007	6.664
303	Cyclamic acid	$C_6H_{13}NO_3S$	C02824	180.1018	0.556
304	Galactosamine	$C_6H_{13}NO_5$	C02262	180.0868	0.859473876
305	Metanephrine	$C_{10}H_{15}NO_3$	C05588	180.1024	2.779418264
306	3,6-Dihydroxypyridine-2,5-dione	$C_5H_3NO_4$	C22287	180.1024	7.546921033
307	2-Amino-4,6-dinitrotoluene	$C_7H_7N_3O_4$	C16395	181.0483	0.604147623
308	3-Hydroxyl kyneurenine	$C_{10}H_{12}N_2O_4$	C02794	181.0972	0.278
309	4-Amino-2,6-dinitrotoluene	$C_7H_7N_3O_4$	C16394	181.0483	0.604147623
310	L-3-Hydroxykynurenine	$C_{10}H_{12}N_2O_4$	C03227	181.0971	0.274
311	Ricinine	$C_8H_8N_2O_2$	C01526	182.1173	1.376
312	4-Isopropylbenzoic acid	$C_{10}H_{12}O_2$	C06578	182.1179	1.918
313	Tyrosine	$C_9H_{11}NO_3$	C01536	182.081	0.912
314	62641-07-0	$C_{10}H_{14}O_4$	C06579	182.1453	14.282
315	4-Hydroxycinnamic acid	$C_9H_8O_3$	C00811	182.081	0.918
316	L-Sorbitol	$C_6H_{14}O_6$	C01722	183.0652	7.744
317	3,4-Dihydroxyhydrocinnamic acid	$C_9H_{10}O_4$	C10447	183.0666	7.744
318	Chorismate	$C_{10}H_{10}O_6$	C00251	183.0654	1.188536446
319	Monobenzone	$C_{13}H_{12}O_2$	C14244	183.0804	0.2
320	Diisopropylphosphate	$C_6H_{15}O_4P$	C03113	183.0655	1.735
321	Homovanillic Acid	$C_9H_{10}O_4$	C05582	183.0851	6.613
322	CHEMBL1161513	$C_9H_{13}NO_4$	C20941	183.0654	1.195
323	3-(4-hydroxyphenyl)lactate	$C_9H_{10}O_4$	C03672	183.0849	7.706
324	5-chlorobenzene-1,2,4-triol	$C_6H_5ClO_3$	C07102	182.984	11.24852391
325	Dihydroclavaminate	$C_8H_{12}N_2O_4$	C06659	184.0605	0.376940734

326	Harmolol	$C_{12}H_{12}N_2O$	C06537	184.097	13.55057443
327	8-oxogeranial	$C_{10}H_{14}O_2$	C17622	184.1333	0.544061829
328	1,6,6-Trimethyl-2,7-dioxabicyclo[3.2.2]nonan-3-one	$C_{10}H_{16}O_3$	C04718	185.1173	0.459059742
329	Choline phosphate	$[C_5H_{15}NO_4P]^+$	C00588	185.0809	1.309806144
330	Dibenzothiophene	$C_{12}H_8S$	C20125	185.0445	13.80630011
331	Ureidoperacrylic acid	$C_4H_6N_2O_4$	C20231	185.1294	5.979709868
332	Carbazole	$C_{12}H_9N$	C08060	185.1075	0.967977527
333	2-Aminobiphenyl-2,3-diol	$C_{12}H_{11}NO_2$	C08061	185.081	13.56714141
334	Pyridoxal	$C_8H_9NO_3$	C00250	185.0918	1.436152763
335	threo-b-methylaspartate	$C_5H_9NO_4$	C03618	186.1488	10.781
336	5-Phosphoribosylamine	$C_5H_{12}NO_7P$	C03090	186.055	12.98744941
337	Spermine	$C_{10}H_{26}N_4$	C00750	186.2218	6.594
338	10-oxocapric acid	$C_{10}H_{18}O_3$	C02217	187.1327	0.878
339	Benzyl isothiocyanate	C_8H_7NS	C03098	188.1282	2.819247194
340	PROPAMOCARB	$C_9H_{20}N_2O_2$	C18885	189.1597	0.245
341	Cyromazine	$C_6H_{10}N_6$	C14147	189.0868	4.70313052
342	Menadione	$C_{11}H_8O_2$	C05377	190.0871	4.459954936
343	Perillyl alcohol	$C_{10}H_{16}O$	C02452	191.1431	10.428
344	5-Hydroxyindoleacetic acid	$C_{10}H_9NO_3$	C05635	192.0665	5.119
345	Toxoflavin	$C_7H_7N_5O_2$	C16789	194.0648	12.61753857
346	4-Nitrocatechol	$C_6H_5NO_4$	C02235	194.1172	7.255476818
347	1,3,7-trimethylurate	$C_8H_{10}N_4O_3$	C16361	194.0801	1.425081191
348	3-Amino-4,7-dihydroxycoumarin	$C_9H_7NO_4$	C12468	194.0448	0.08956643
349	5,6-dihydroxy-3-methyl-5,6-dihydroquinolin-2(1H)-one	$C_{10}H_{11}NO_3$	C06726	194.0808	1.885916072
350	D-Pinitol	$C_7H_{14}O_6$	C03844	195.0866	1.482315546
351	FA 12_2;O	$C_{12}H_{20}O_3$	C16311	195.138	0.264

352	3-Dehydro-L-gulonate	$C_6H_{10}O_7$	C00618	195.0653	0.603
353	Harmine	$C_{13}H_{12}N_2O$	C06538	196.0981	7.11184912
354	2,5-dichlorohydroquinone	$C_6H_4Cl_2O_2$	C06600	195.9932	2.756115518
355	3,5-DICHLOROCATECHOL	$C_6H_4Cl_2O_2$	C02933	195.9928	0.715229845
356	Coniferaldehyde	$C_{10}H_{10}O_3$	C02666	196.0969	0.426217344
357	N-alpha-acetylornithine	$C_7H_{14}N_2O_3$	C00437	197.0921	2.999
358	phenprobamate	$C_{10}H_{13}NO_2$	C76122	197.1273	5.83
359	2-Hexenal	$C_6H_{10}O$	C08497	197.1537	0.515232024
360	Dihydropinosylvin	$C_{14}H_{14}O_2$	C10254	197.1003	0.071944092
361	Harmaline	$C_{13}H_{14}N_2O$	C06536	198.1132	9.811663736
362	Caffeic acid	$C_9H_8O_4$	C01197	198.1239	1.016
363	4-chloro-L-lysine	$C_6H_{13}ClN_2O_2$	C22137	198.0974	15.024
364	Deoxyribose 1-phosphate	$C_5H_{11}O_7P$	C00672	198.0029	10.43429212
365	L-Dopa	$C_9H_{11}NO_4$	C00355	198.0765	2.115
366	L-2-Amino-3-(4-aminophenyl)propanoic acid	$C_9H_{12}N_2O_2$	C12033	198.1126	0.678
367	Naphthalene-1,2-diol	$C_{10}H_8O_2$	C03012	199.1522	10.15193355
368	Ethoprophos	$C_8H_{19}O_2PS_2$	C18687	199.1065	12.736
369	D-Alanyl-D-alanine	$C_6H_{12}N_2O_3$	C00993	199.1805	9.737997074
370	2-C-methyl-D-erythritol-4-phosphate	$C_5H_{13}O_7P$	C11434	199.1068	4.586
371	Bergapten	$C_{12}H_8O_4$	C01557	200.047	1.033656591
372	Neryl formate	$C_{11}H_{18}O_2$	C12294	200.0919	0.843
373	Gamma-glutamyl-L-putrescine	$C_9H_{19}N_3O_3$	C15699	201.1474	1.139362975
374	Camalexin	$C_{11}H_8N_2S$	C21721	201.051	14.45792311
375	Uridine	$C_9H_{12}N_2O_6$	C00299	201.0871	0.598645562
376	Isoxanthopterin	$C_6H_5N_5O_2$	C03975	202.071	5.31
377	L-Coprine	$C_8H_{14}N_2O_4$	C08271	203.139	0.06
378	Proclavaminate	$C_8H_{14}N_2O_4$	C06658	203.1025	0.634
379	Isoeugenol	$C_{10}H_{12}O_2$	C10469	203.1835	9.958386877

380	2,4-diacetamido-2,4,6-trideoxy-beta-L-gulopyranose	$C_{10}H_{18}N_2O_5$	C22254	203.139	0.060156346
381	Homoserine, O-succinyl-	$C_8H_{13}NO_6$	C01118	203.0815	13.19558847
382	N-Acetyl-D-glucosamine	$C_8H_{15}NO_6$	C00140	204.0877	5.172
383	SCHEMBL9621780	$C_7H_6O_6$	C04480	204.0494	4.229
384	Pyrimidodiazepine	$C_9H_{11}N_5O_2$	C02587	204.0852	7.077
385	3-Phenylcatechol	$C_{12}H_{10}O_2$	C02526	204.1026	3.422689863
386	N-Acetylmannosamine	$C_8H_{15}NO_6$	C00645	204.0865	0.708
387	Linamarin	$C_{10}H_{17}NO_6$	C01594	204.1231	0.349690456
388	N-(3-Methylbut-2-EN-1-YL)-9H-purin-6-amine	$C_{10}H_{13}N_5$	C04083	204.1232	5.223412026
389	9-methylthiononanaldoxime	$C_{10}H_{21}NOS$	C17253	204.1425	4.144065543
390	N-Acetyl-b-glucosaminylamine	$C_8H_{16}N_2O_5$	C01239	204.0865	0.70568166
391	Indolepyruvate	$C_{11}H_9NO_3$	C00331	204.0656	0.407613532
392	coniferyl acetate	$C_{12}H_{14}O_4$	C20225	205.0861	0.898061351
393	5-Methoxyindoleacetate	$C_{11}H_{11}NO_3$	C05660	206.0814	1.135376118
394	Levonordefrin	$C_9H_{13}NO_3$	C11768	206.0827	18.636
395	MPTP N-oxide	$C_{12}H_{15}NO$	C04731	207.1493	0.563
396	Uric acid	$C_5H_4N_4O_3$	C00366	207.1241	9.611726013
397	Limonene-1,2-diol	$C_{10}H_{18}O_2$	C07276	209.1538	8.881
398	Porphobilinogen	$C_{10}H_{14}N_2O_4$	C00931	209.092	1.608
399	naphthalene-1,2,4,8-tetrol	$C_{10}H_8O_4$	C21414	210.076	0.391382642
400	trans-Coumaryl acetate	$C_{11}H_{12}O_3$	C20465	210.1123	0.788245619
401	Nitroglycerin	$C_3H_5N_3O_9$	C07455	210.0953	18.872
402	(2E,5S,6E,8E,10E)-5-hydroxydodeca-2,6,8,10-tetraenal	$C_{12}H_{16}O_2$	C22087	210.149	0.718442153
403	beta-Ionone	$C_{13}H_{20}O$	C12287	210.1852	0.154245399

404	1,3,7-trimethyl-5-hydroxyisourate	$C_8H_{10}N_4O_4$	C21144	210.0762	6.957379751
405	2,5-Diketogluconsaure	$C_6H_8O_7$	C02780	210.0606	1.081688332
406	Aprobarbital	$C_{10}H_{14}N_2O_3$	C07826	211.1689	1.646
407	6-Hydroxymellein	$C_{10}H_{10}O_4$	C02379	212.0911	3.44861451
408	Deoxyuridine	$C_9H_{12}N_2O_5$	C00526	212.0554	0.256
409	Deoxyamidinoproclavaminate	$C_9H_{16}N_4O_3$	C06656	212.103	0.179063474
410	5-Methoxytryptamine	$C_{11}H_{14}N_2O$	C05659	213.1017	8.784068358
411	m-chlorophenylpiperazine (m-CPP)	$C_{10}H_{13}ClN_2$	C11738	214.1068	17.964
412	Chlorpropham	$C_{10}H_{12}ClNO_2$	C14506	214.0629	0.134
413	Fenchyl acetate	$C_{12}H_{20}O_2$	C11338	214.1802	0.245
414	4-ethylamino-6-isopropylamino-1,3,5-triazin-2-ol	$C_8H_{15}N_5O$	C06552	215.1643	13.1089586
415	Luvangetin	$C_{15}H_{14}O_4$	C09273	215.1027	18.367
416	Amifostine	$C_5H_{15}N_2O_3PS$	C06819	215.139	0.057
417	4-(Glutamylamino) butanoate	$C_9H_{16}N_2O_5$	C15767	215.1016	4.779
418	Methyl 8-methyl-3-oxo-8-azabicyclo[3.2.1]octane-2-carboxylate	$C_{10}H_{15}NO_3$	C20371	215.1391	0.402
419	Glucosamine 1-phosphate	$C_6H_{14}NO_8P$	C06156	216.0629	1.146981273
420	Melatonin	$C_{13}H_{16}N_2O_2$	C01598	216.1231	12.06728989
421	O-Phosphohomoserine	$C_4H_{10}NO_6P$	C01102	217.0609	11.5247836
422	gamma-Glutamyl-gamma-aminobutyraldehyde	$C_9H_{16}N_2O_4$	C15700	217.1182	2.201662416
423	Galacturonic acid	$C_6H_{10}O_7$	C08348	217.1545	8.183
424	D-Erythrose 4-phosphate	$C_4H_9O_7P$	C00279	218.0447	10.48032766
425	Lotaustralin	$C_{11}H_{19}NO_6$	C08334	218.1386	0.356746124
426	Geranyl Phosphate	$C_{10}H_{19}O_4P$	C21203	218.081	8.335

427	Securinine	$C_{13}H_{15}NO_2$	C10614	218.1176	0.232
428	Angustifoline	$C_{14}H_{22}N_2O$	C10751	218.1539	0.145
429	beta-Alanyl-L-lysine	$C_9H_{19}N_3O_3$	C05341	218.15	0.409
430	Reumycin	$C_6H_5N_5O_2$	C21957	218.1397	10.88486002
431	N-Acetylserotonin	$C_{12}H_{14}N_2O_2$	C00978	219.1132	1.831
432	Galactitol 1-phosphate	$C_6H_{15}O_9P$	C06311	219.0613	6.90866027
433	1,2,4-Trichlorobenzene	$C_6H_3Cl_3$	C06594	219.0266	5.406741008
434	L-Lysopine	$C_9H_{18}N_2O_4$	C04020	219.1339	0.124
435	alpha-Curcumene	$C_{15}H_{22}$	C09649	220.2037	10.29328799
436	L-pentahomomethionine	$C_{10}H_{21}NO_2S$	C17229	220.1365	0.314
437	N-Acetyl-S-(N-methylcarbamoyl)cysteine	$C_7H_{12}N_2O_4S$	C11490	221.0768	11.278
438	2,6-Dioxo-6-phenylhexanoate	$C_{12}H_{12}O_4$	C03750	221.0928	2.101
439	Didehydroagroclavine	$C_{16}H_{17}N_2+$	C20457	221.121	5.003504416
440	Fraxidin	$C_{11}H_{10}O_5$	C17479	223.0601	0.012
441	Diethyl phthalic acid	$C_{12}H_{14}O_4$	C14175	223.0962	1.259
442	Dihydrobiopterin	$C_9H_{13}N_5O_3$	C02953	223.1074	4.611141988
443	2-Amino-3-carboxymuconic acid semialdehyde	$C_7H_7NO_5$	C04409	224.1287	6.570421816
444	Scoparone	$C_{11}H_{10}O_4$	C09311	224.0917	0.140210905
445	Methylisocitric acid	$C_7H_{10}O_7$	C04593	224.1288	0.38
446	Tetrahydrobiopterin	$C_9H_{15}N_5O_3$	C00272	224.1167	11.17533812
447	Phosphoserine	$C_3H_8NO_6P$	C01005	224.1071	1.895432586
448	Methyl jasmonate	$C_{13}H_{20}O_3$	C11512	225.1486	0.385
449	1,3-dihydroxy-N-methylacridone	$C_{14}H_{11}NO_3$	C12093	225.0762	9.890073229
450	1-Methoxyphenanthrene	$C_{15}H_{12}O$	C11433	226.1224	1.042
451	beta-Caryophyllene	$C_{15}H_{24}$	C09629	227.1755	6.652
452	2-heptylquinolin-4(1H)-one	$C_{16}H_{21}NO$	C20643	227.1642	11.63044221

453	L-tetrahomomethionine	$C_9H_{19}NO_2S$	C17225	228.1056	11.99400585
454	Diaminopimelic acid	$C_7H_{14}N_2O_4$	C00666	229.1544	10.126
455	Perseitol	$C_7H_{16}O_7$	C08255	230.1195	17.052
456	Curzerenone	$C_{15}H_{18}O_2$	C16943	231.17	1.344
457	Naproxen	$C_{14}H_{14}O_3$	C01517	231.0609	18.533
458	Abscisic alcohol	$C_{15}H_{22}O_3$	C13456	233.1399	16.717
459	Pinosylvin	$C_{14}H_{12}O_2$	C01745	235.0713	7.001705865
460	Gluconic acid	$C_6H_{12}O_7$	C00257	235.1542	7.874067739
461	Lidocaine	$C_{14}H_{22}N_2O$	C07073	235.1808	1.358
462	phlorisobutyrophenone	$C_{10}H_{12}O_4$	C07351	235.1695	7.682204112
463	Precocene II	$C_{13}H_{16}O_3$	C09018	238.0712	0.852
464	Daidzein	$C_{15}H_{10}O_4$	C10208	238.0378	3.501209052
465	2-Amino-6-[(1R,2S)-1,2,3-trihydroxypropyl]-7,8-dihydro-3H-pteridin-4-one	$C_9H_{13}N_5O_4$	C04874	239.1029	6.74931128
466	Machiline	$C_{17}H_{19}NO_3$	C06348	242.1534	2.2
467	Norbelladine	$C_{15}H_{17}NO_3$	C16701	242.1172	1.439881595
468	6-Phosphonoglucono-D-lactone	$C_6H_{11}O_9P$	C01236	241.9989	16.959
469	Glycineamideribotide	$C_7H_{15}N_2O_8P$	C03838	243.0761	8.448300347
470	8-hydroxy-alpha-humulene	$C_{15}H_{24}O$	C20229	243.1701	7.509558535
471	Lumichrome	$C_{12}H_{10}N_4O_2$	C01727	243.0883	2.68
472	Lapachol	$C_{15}H_{14}O_3$	C10366	243.0865	4.724
473	Thymidine	$C_{10}H_{14}N_2O_5$	C00214	243.0973	1.002973295
474	N-acetyl-alpha-D-glucosamine	$C_8H_{15}NO_6$	C13970	244.0805	5.515802778
475	Carisoprodol	$C_{12}H_{24}N_2O_4$	C07927	244.0967	16.01
476	Leucyl-leucine	$C_{12}H_{24}N_2O_3$	C11332	245.1862	0.98
477	Deoxycytidine	$C_9H_{13}N_3O_4$	C00881	245.1258	5.600307679
478	1-(2-aminophenyl)decane-1,3-dione	$C_{16}H_{23}NO_2$	C21490	245.1743	12.66943599

479	(-)-alpha-Bisabolol	$C_{15}H_{26}O$	C09621	245.1006	1.188
480	N-Succinyl-L,L-2,6-diaminopimelate	$C_{11}H_{18}N_2O_7$	C04421	247.1325	14.80088576
481	Geroquinol	$C_{16}H_{22}O_2$	C10793	247.1661	11.92565532
482	Acetyl-N-formyl-5-methoxykynurenamine	$C_{13}H_{16}N_2O_4$	C05642	247.1112	11.68294642
483	Pentalenolactone D	$C_{15}H_{20}O_4$	C20399	247.1288	16.038
484	6-HYDROXYMELATONIN	$C_{13}H_{16}N_2O_3$	C05643	249.1236	0.951254317
485	Sinapyl alcohol	$C_{11}H_{14}O_4$	C02325	249.189	8.203331206
486	4,4-dihydroxy-alpha-methylstilbene	$C_{15}H_{14}O_2$	C13632	249.0872	5.199382305
487	Multifidol	$C_{11}H_{14}O_4$	C10709	249.1851	7.447556455
488	Cordycepin	$C_{10}H_{13}N_5O_3$	C08431	252.1074	6.788
489	5-Deoxy-5-fluoroadenosine	$C_{10}H_{12}FN_5O_3$	C19766	252.09	3.471696219
490	Deoxyadenosine	$C_{10}H_{13}N_5O_3$	C00559	252.1087	1.631
491	Inosine	$C_{10}H_{12}N_4O_5$	C00294	252.0821	12.70467082
492	demethylsuberosin	$C_{14}H_{14}O_3$	C18083	253.0809	10.31330337
493	Tetaine	$C_{12}H_{18}N_2O_5$	C20942	253.1185	0.88
494	(R)-Higenamine	$C_{16}H_{17}NO_3$	C06346	254.1217	16.336
495	3-Dimethylallyl-4-hydroxymandelic acid	$C_{13}H_{16}O_4$	C12457	254.1014	2.738
496	Kinetin	$C_{10}H_9N_5O$	C08272	254.183	17.87
497	N2-Succinyl-L-glutamic acid 5-semialdehyde	$C_9H_{13}NO_6$	C05932	254.0659	9.428655715
498	FAL 16_1	$C_{16}H_{30}O$	C06123	256.2274	1.19
499	Palmitaldehyde	$C_{16}H_{32}O$	C00517	258.2795	1.442
500	Zeatin	$C_{10}H_{13}N_5O$	C00371	258.2063	13.38549873
501	Glycerophosphocholine	$C_8H_{20}NO_6P$	C00670	258.1104	1.18
502	Gamma-glutamylglutamine	$C_{10}H_{17}N_3O_6$	C05283	259.1129	11.835
503	epsilon-(gamma-Glutamyl)lysine	$C_{11}H_{21}N_3O_5$	C21730	259.1288	0.158
504	Saccharopine	$C_{11}H_{20}N_2O_6$	C00449	259.1186	0.932

505	Galactose 1-phosphate	$C_6H_{13}O_9P$	C00446	261.0368	0.744
506	Mannose 6-phosphate	$C_6H_{13}O_9P$	C00275	261.0368	0.744
507	L-Cystathionine	$C_7H_{14}N_2O_4S$	C02291	261.1638	5.268035233
508	Alcoifosfamide	$C_7H_{17}Cl_2N_2O_3P$	C16558	262.0404	1.845
509	N1-(alpha-D-ribosyl)-5,6-dimethyl-benzimidazole	$C_{14}H_{18}N_2O_4$	C05775	262.128	12.15902193
510	1,2-Bis(4-hydroxyphenyl)-2-propanol	$C_{15}H_{16}O_3$	C13629	262.1472	13.10706313
511	cyclopeptine	$C_{17}H_{16}N_2O_2$	C20579	263.1221	16.027
512	trans-2,3-Epoxysuccinate	$C_4H_4O_5$	C03548	265.0177	4.993
513	2-(6-methylthio)hexylmalate	$C_{11}H_{20}O_5S$	C17226	265.1106	0.696
514	3-(6-methylthio)hexylmalate	$C_{11}H_{20}O_5S$	C17227	265.111	2.205
515	Artemisinin	$C_{15}H_{22}O_5$	C09538	266.1516	1.30519561
516	Thiamine	$C_{12}H_{17}N_4OS$	C00378	266.1154	15.698
517	L-Glutamic acid 5-phosphate	$C_5H_{10}NO_7P$	C03287	266.1136	13.67618979
518	Amidinoproclavaminate	$C_9H_{16}N_4O_4$	C06657	267.1073	3.48062333
519	gamma-Glutamylcysteine	$C_8H_{14}N_2O_5S$	C00669	268.1002	15.05101414
520	Xanthosine	$C_{10}H_{12}N_4O_6$	C01762	268.0848	17.093
521	Adenosine	$C_{10}H_{13}N_5O_4$	C00212	268.1036	1.59
522	beta-Alanyl-L-arginine	$C_9H_{19}N_5O_3$	C05340	268.1368	1.13
523	Arborinine	$C_{16}H_{15}NO_4$	C10643	269.1018	10.551
524	Caranine	$C_{16}H_{17}NO_3$	C08521	272.1276	1.887423768
525	(-)-Sabinene	$C_{10}H_{16}$	C16777	273.2533	15.969
526	Dihydrotestosterone	$C_{19}H_{30}O_2$	C03917	273.225	13.617
527	Sarmentosin	$C_{11}H_{17}NO_7$	C08340	276.1568	9.458

528	5-Amino-6-ribitylamino uracil	$C_9H_{16}N_4O_6$	C04732	277.1182	14.232
529	corynantheal	$C_{19}H_{22}N_2O$	C16735	278.1564	8.874071925
530	Sulfamethazine	$C_{12}H_{14}N_4O_2S$	C19530	279.0927	6.024
531	Dibutyl phthalate	$C_{16}H_{22}O_4$	C14214	279.1591	0.08
532	Diisobutyl phthalate	$C_{16}H_{22}O_4$	C15205	279.0927	6.024
533	Pulcherriminic acid	$C_{12}H_{20}N_2O_4$	C20515	279.1272	19.63
534	17a-Ethynylestradiol	$C_{20}H_{24}O_2$	C07534	279.1773	10.632
535	Prunasin	$C_{14}H_{17}NO_6$	C00844	279.0822	14.72548267
536	Disulfiram	$C_{10}H_{20}N_2S_4$	C01692	279.1004	9.878
537	Pantetheine	$C_{11}H_{22}N_2O_4S$	C00831	279.134	11.814
538	L-4-Hydroxyphenylglycyl-L-arginine	$C_{14}H_{21}N_5O_4$	C20910	280.1384	18.568
539	Phaseollidin	$C_{20}H_{20}O_4$	C05230	281.142	14.9330573
540	N-Glycolylneuraminic acid	$C_{11}H_{19}NO_{10}$	C03410	282.1522	11.881
541	2-Heptyl-3-hydroxy-quinolone	$C_{16}H_{21}NO_2$	C11848	282.1465	0.208047592
542	Oleamide	$C_{18}H_{35}NO$	C19670	282.2797	2.032
543	7-Methylxanthosine	$C_{11}H_{15}N_4O_6+$	C16352	282.0973	5.081863244
544	Nandinine	$C_{19}H_{19}NO_4$	C21370	282.1533	15.77929409
545	1-(4-Hydroxyphenyl)-1-decene-3,5-dione	$C_{16}H_{20}O_3$	C17744	283.1284	5.854932808
546	Sphinganine	$C_{18}H_{39}NO_2$	C00836	284.2951	1.144
547	Octadecanamide	$C_{18}H_{37}NO$	C13846	284.2947	0.266
548	Guanosine	$C_{10}H_{13}N_5O_5$	C00387	284.099	0.207
549	Salidroside	$C_{14}H_{20}O_7$	C06046	284.1236	6.452896204
550	(S)-scoulerine	$C_{19}H_{21}NO_4$	C02106	284.1604	14.417984
551	Capsaicin	$C_{18}H_{27}NO_3$	C06866	288.1913	15.596
552	Tetraphyllin B	$C_{12}H_{17}NO_7$	C08344	288.2533	0.025
553	ascopyrone P	$C_6H_8O_4$	C19826	289.0926	2.802
554	Maleic acid homopolymer	$C_6H_8O_4$	C00922	289.0917	0.311
555	2-Naphthyl beta-D-glucopyranoside	$C_{16}H_{18}O_6$	C75823	290.1128	5.064295893
556	6,7,12,13-Tetrahydro-5H-	$C_{20}H_{13}N_3O$	C21126	294.1178	6.374

	indolo[2,3-a]pyrrolo[3,4-c]carbazol-5-one				
557	Taxiphyllin	$C_{14}H_{17}NO_7$	C01855	294.1009	12.55140634
558	(-)-Thebaine	$C_{19}H_{21}NO_3$	C06173	294.1547	19.898
559	Machete	$C_{17}H_{26}ClNO_2$	C10931	295.1476	5.678446648
560	Estradiol	$C_{18}H_{24}O_2$	C00951	295.1647	7.253916542
561	Aspartame	$C_{14}H_{18}N_2O_5$	C11045	295.1284	1.498
562	9JHS2AVR43	$C_{16}H_{16}O_4$	C16225	295.0924	5.673816405
563	9(S)-HPODE	$C_{18}H_{32}O_4$	C14827	295.2264	1.215
564	juvenile hormone I	$C_{18}H_{30}O_3$	C19613	295.2268	1.831
565	Demethylmedicarpin	$C_{15}H_{12}O_4$	C04271	295.1738	8.447
566	8(R)-Hydroperoxylinoleic acid	$C_{18}H_{32}O_4$	C14831	296.2189	18.381
567	9,10-DiHOME	$C_{18}H_{34}O_4$	C14828	297.2902	9.621
568	5-methylthioadenosine (MTA)	$C_{11}H_{15}N_5O_3S$	C00170	298.0965	1.115
569	(+)-Larreatricin	$C_{18}H_{20}O_3$	C20430	302.2073	13.718
570	(-)-Maackiain	$C_{16}H_{12}O_5$	C10502	302.1003	6.605819657
571	10beta-hydroxytaxa-4(20),11-dien-5alpha-yl acetate	$C_{22}H_{34}O_3$	C11898	303.2637	14.93030686
572	SCHEMBL4290912	$C_{16}H_{21}N_3O_3$	C04211	304.1646	3.156
573	Nicotianamine	$C_{12}H_{21}N_3O_6$	C05324	304.1494	17.185
574	ST 18_4;O3	$C_{18}H_{22}O_3$	C05298	304.1871	11.873
575	10-Hydroxydihydrosanguinarine	$C_{20}H_{15}NO_5$	C05247	306.1447	13.548
576	Difructose anhydride III	$C_{12}H_{20}O_{10}$	C04420	307.1018	1.802
577	Biphenyl	$C_{12}H_{10}$	C06588	309.165	3.983568321
578	6,7-Dimethyl-8-(1-D-ribityl)lumazine	$C_{13}H_{18}N_4O_6$	C04332	310.1284	3.981511851
579	5-Nitro-2-(3-phenylpropylamino)benzoic acid	$C_{16}H_{16}N_2O_4$	C13705	318.0972	0.033

580	9alpha-Hydroxyandrosta-1,4-diene-3,17-dione	$C_{19}H_{24}O_3$	C14909	318.2022	13.07602556
581	4-O-(Indole-3-acetyl)-D-glucopyranose	$C_{16}H_{19}NO_7$	C04197	321.1178	8.977
582	Oryzalexin E	$C_{20}H_{32}O_2$	C21561	322.2734	1.994021536
583	protodeoxyviolaceinic acid	$C_{21}H_{15}N_3O_2$	C21131	324.1289	0.004
584	Corydalis L	$C_{20}H_{23}NO_4$	C04118	324.1552	12.991
585	Uridine 5'-monophosphate	$C_9H_{13}N_2O_9P$	C00105	325.048	14.94480784
586	Mycocyclosin	$C_{18}H_{16}N_2O_4$	C20517	325.1131	0.866
587	3,6-anhydro-alpha-L-galactopyranose	$C_6H_{10}O_5$	C20902	325.1132	0.866
588	Phaseollidin hydrate	$C_{20}H_{22}O_5$	C05229	326.1497	4.760452332
589	Stanozolol	$C_{21}H_{32}N_2O$	C07311	329.2319	1.029
590	S-(Indolylmethylthiohydroximoyl)-L-cysteine	$C_{13}H_{15}N_3O_3S$	C16518	332.1858	13.96983224
591	Pyridoxine O-glucoside	$C_{14}H_{21}NO_8$	C03996	332.1318	6.585
592	5a-Pregnane-3,20-dione	$C_{21}H_{32}O_2$	C03681	334.2735	1.623281833
593	15-Keto-prostaglandin F2a	$C_{20}H_{32}O_5$	C05960	336.3113	10.525
594	Oxymesterone	$C_{20}H_{30}O_3$	C14665	336.2893	1.165
595	3-Dehydrosphinganine	$C_{18}H_{37}NO_2$	C02934	338.3777	7.287
596	Genomorphine	$C_{17}H_{19}NO_4$	C11786	340.3936	6.507
597	4-Hydroxysphinganine	$C_{18}H_{39}NO_3$	C12144	340.2849	7.937172352
598	2,1 dianhydride	$C_{12}H_{20}O_{10}$	C04333	342.1393	0.782
599	ST 19_2;O3	$C_{19}H_{28}O_3$	C14671	343.2941	7.021
600	Dibucaine	$C_{20}H_{29}N_3O_2$	C07879	344.228	15.228
601	N-Didesmethyl-tamoxifen	$C_{24}H_{25}NO$	C16548	344.2265	13.047

602	Combretum caffrum	$C_{17}H_{12}O_8$	C10836	345.1016	13.692
603	Neochlorogenic acid	$C_{16}H_{18}O_9$	C17147	355.1011	3.532
604	(-)-Aspidospermine	$C_{22}H_{30}N_2O_2$	C09042	355.1152	5.169
605	Gentiopicrin	$C_{16}H_{20}O_9$	C09782	357.2126	18.406
606	Isocolumbin	$C_{20}H_{22}O_6$	C09077	358.9791	6.399
607	4-O-beta-D-Mannopyranosyl-D-mannopyranose	$C_{12}H_{22}O_{11}$	C20861	360.1478	11.85153114
608	Lactulose	$C_{12}H_{22}O_{11}$	C07064	360.147	8.418
609	Roquefortine L	$C_{22}H_{21}N_5O_3$	C22170	360.1834	4.225569252
610	SCHEMBL12391563	$C_{19}H_{18}O_6$	C18678	360.1477	6.474
611	4-O-beta-D-mannopyranosyl-D-glucopyranose	$C_{12}H_{22}O_{11}$	C20236	360.1478	11.85153114
612	10,11-Epoxy-3-geranylgeranylindole	$C_{28}H_{39}NO$	C20526	362.3213	1.946283313
613	Aurachin D	$C_{25}H_{33}NO$	C10641	364.2671	10.22
614	Sphinganine 1-phosphate	$C_{18}H_{40}NO_5P$	C01120	365.2696	0.732555332
615	Coniferin	$C_{16}H_{22}O_8$	C00761	365.1026	5.278
616	Tetrahydrocortisone	$C_{21}H_{32}O_5$	C05470	365.191	13.295
617	2-Hydroxy-6-pentadecylbenzoic acid	$C_{22}H_{36}O_3$	C10759	366.2996	1.791
618	Aflatoxin Q1	$C_{17}H_{12}O_7$	C19585	367.1496	17.572
619	isoroquefortine C	$C_{22}H_{23}N_5O_2$	C22165	373.1849	12.86820581
620	Deoxycorticosterone acetate	$C_{23}H_{32}O_4$	C14554	373.2383	2.616
621	Karacoline	$C_{22}H_{35}NO_4$	C08693	378.2606	8.649
622	Aurachin B	$C_{25}H_{33}NO_2$	C21140	380.2599	3.965
623	Ergosterol	$C_{28}H_{44}O$	C01694	380.3367	18.493
624	glandicoline A	$C_{22}H_{21}N_5O_3$	C22166	387.1404	12.29274987
625	ST 24_3;O5	$C_{24}H_{36}O_5$	C15568	388.2535	18.794
626	Hydrocortisoni acetas	$C_{23}H_{32}O_6$	C02821	388.2027	5.398
627	gamma-Tocotrienol	$C_{28}H_{42}O_2$	C14155	393.3166	3.624
628	Clovamide	$C_{18}H_{17}NO_7$	C00014	398.2012	7.412

629	Tris(2-butoxyethyl) phosphate	$C_{18}H_{39}O_7P$	C14446	399.2483	5.77
630	Rosmarinic acid	$C_{18}H_{16}O_8$	C01850	399.177	13.19
631	Pravastatin	$C_{23}H_{36}O_7$	C01844	407.2373	11.546
632	Cycloeucalenone	$C_{30}H_{48}O$	C22121	408.3692	14.282
633	ABA-GE cpd	$C_{21}H_{30}O_9$	C15970	409.1836	5.094094925
634	Fenobucarb	$C_{12}H_{17}NO_2$	C14425	415.267	18.974
635	Chlorimuron ethyl	$C_{15}H_{15}ClN_4O_6S$	C10943	415.0423	12.183
636	27-Deoxy-5b-cyprinol	$C_{27}H_{48}O_4$	C05446	420.3318	9.916499537
637	Demethylphylloquinone	$C_{30}H_{44}O_2$	C13309	420.3431	10.58939685
638	Lactose 6-phosphate	$C_{12}H_{23}O_{14}P$	C05396	423.0879	4.526766424
639	Trehalose 2-sulfate	$C_{12}H_{22}O_{14}S$	C20985	423.087	15.837
640	Licoricidin	$C_{26}H_{32}O_5$	C16986	425.2135	15.079
641	atrazine	$C_8H_{14}ClN_5$	C06551	431.1895	12.31760073
642	Gentamicinc1A	$C_{19}H_{39}N_5O_7$	C00908	432.2795	4.964
643	Glycyrrhetol	$C_{30}H_{48}O_3$	C20510	440.358	15.583
644	1,6-di-O-Galloylglucose	$C_{20}H_{20}O_{14}$	C04101	441.0992	8.044494526
645	4'-Phosphopantothenoylcysteine	$C_{12}H_{23}N_2O_9PS$	C04352	441.1863	5.360048351
646	Roquefortine F	$C_{23}H_{25}N_5O_3$	C22171	442.1905	12.54956834
647	omega-Hydroxyphylloquinone	$C_{31}H_{46}O_3$	C20806	449.3432	4.033
648	ST 28_0;O5	$C_{28}H_{50}O_5$	C15803	450.3675	6.309114445
649	ST 28_1;O3	$C_{28}H_{48}O_3$	C15800	450.39	9.216945536
650	5-Methyltetrahydrofolic acid	$C_{20}H_{25}N_7O_6$	C00440	460.2001	13.47366048
651	Vitamin K	$C_{31}H_{46}O_2$	C02059	468.3798	11.34309524
652	Phylloquinol	$C_{31}H_{48}O_2$	C03313	470.3947	10.28120254
653	Citicoline	$C_{14}H_{26}N_4O_{11}P_2$	C00307	471.1039	0.292971678
654	Biopterin	$C_9H_{11}N_5O_3$	C06313	475.1746	10.623
655	Terpendole I	$C_{27}H_{35}NO_5$	C20543	476.3045	13.138
656	2-Methoxy-estradiol-17b 3-glucuronide	$C_{25}H_{34}O_9$	C11131	479.2734	12.073

657	Aflatrem	$C_{32}H_{39}NO_4$	C20555	485.2884	8.303969557
658	Aquayamycin	$C_{25}H_{26}O_{10}$	C12412	487.1631	7.976
659	Cytidine	$C_9H_{13}N_3O_5$	C00475	487.1768	3.099532038
660	alpha-D-Glucopyranosyl-(1->4)-alpha-D-glucopyranosyl-(1->6)-D-glucose	$C_{18}H_{32}O_{16}$	C03367	487.1572	17.527
661	Protopanaxatriol	$C_{30}H_{52}O_4$	C20716	499.3699	11.53433346
662	Melezitose	$C_{18}H_{32}O_{16}$	C08243	505.1729	6.935
663	Neurosporene	$C_{40}H_{58}$	C05431	522.4342	8.318254824
664	13-Deoxycarminomycin	$C_{26}H_{29}NO_9$	C12428	522.2098	5.09
665	Maltotriose	$C_{18}H_{32}O_{16}$	C01835	522.1971	11.016
666	Raffinose	$C_{18}H_{32}O_{16}$	C00492	527.1527	10.524
667	TR 1 toxin	$C_{27}H_{33}N_3O_7$	C20045	529.2694	7.058371219
668	2-Hexaprenyl-6-methoxy-1,4-benzoquinone	$C_{37}H_{54}O_3$	C05803	530.3826	10.183
669	Alpha-Cryptoxanthin	$C_{40}H_{56}O$	C15981	536.3889	5.424
670	Formononetin	$C_{16}H_{12}O_4$	C00858	537.1617	13.61448499
671	CMP-8-amino-3,8-dideoxy-beta-D-manno-octulosonate	$C_{17}H_{27}N_4O_{14}P$	C21334	543.1279	10.144
672	Protoporphyrin IX	$C_{34}H_{34}N_4O_4$	C02191	546.2619	1.151133001
673	3-Hexaprenyl-4,5-Dihydroxybenzoic acid	$C_{37}H_{54}O_4$	C05200	546.3868	7.108846517
674	Leprotin	$C_{40}H_{48}$	C15943	546.4125	5.087
675	Methanophenazine	$C_{37}H_{50}N_2O$	C11903	556.4279	3.201816264
676	Proneurosporene	$C_{40}H_{58}$	C19759	556.4442	4.156
677	Chloroxanthin	$C_{40}H_{60}O$	C15892	557.4366	1.048
678	Rhodovibrin	$C_{41}H_{60}O_2$	C15878	568.4657	3.267707973
679	beta-Cryptoxanthin	$C_{40}H_{56}O$	C08591	570.472	8.908
680	Anhydrorhodovibrin	$C_{41}H_{58}O$	C15877	584.4886	10.32146718
681	ACMC-20dgc9	$C_{35}H_{38}N_2O_6$	C06512	600.481	5.858
682	Naringin	$C_{27}H_{32}O_{14}$	C09789	603.1639	7.490335711
683	Tubocurarine	$C_{37}H_{41}N_2O_6^+$	C07547	610.1797	19.036

684	Delphinidin 3,5-diglucoside	$C_{27}H_{31}O_{17}+$	C16312	611.1434	10.726
685	Rutin	$C_{27}H_{30}O_{16}$	C05625	611.1541	10.725
686	Astaxanthin	$C_{40}H_{52}O_4$	C08580	614.4917	2.329
687	Demethyl-desacetyl-rifamycin S	$C_{34}H_{41}NO_{11}$	C14724	622.2627	3.148
688	GDP-D-Rhamnose	$C_{16}H_{25}N_5O_{15}P_2$	C03117	628.1788	1.89
689	Ketospirilloxanthin	$C_{42}H_{58}O_3$	C15884	628.4852	11.026
690	Dicrocin	$C_{32}H_{44}O_{14}$	C19868	636.2794	2.783651176
691	Cyclotetraglucose	$C_{24}H_{40}O_{20}$	C21655	649.211	11.64555145
692	Dihydrosanguinarine	$C_{20}H_{15}NO_4$	C05191	667.2105	4.522680473
693	lychnose	$C_{24}H_{42}O_{21}$	C08242	667.223	9.179
694	1,3-alpha-D-Mannosyl-1,2-alpha-D-mannosyl-1,2-alpha-D-mannosyl-D-mannose	$C_{24}H_{42}O_{21}$	C04861	684.2607	7.2
695	isolychnose	$C_{24}H_{42}O_{21}$	C03989	684.2458	11.94541463
696	Melibiose	$C_{12}H_{22}O_{11}$	C00252	685.2288	1.252
697	Sucrose	$C_{12}H_{22}O_{11}$	C00089	685.2495	14.318
698	Dephospho-CoA	$C_{21}H_{35}N_7O_{13}P_2S$	C00882	705.1751	10.77380653
699	Bacteriohopanetetrol glucosamine	$C_{41}H_{73}NO_8$	C21121	730.5294	9.02
700	5-Methyltetrahydropteroyltri-L-glutamic acid	$C_{30}H_{39}N_9O_{12}$	C04489	756.3799	13.67458324
701	7,8-Dihydromethanopterin	$C_{30}H_{43}N_6O_{16}P$	C05927	758.23	2.595
702	Shisonin	$C_{36}H_{37}O_{18}+$	C12096	758.2138	11.26777157
703	Reduced coenzyme F420	$C_{29}H_{38}N_5O_{18}P$	C01080	758.1977	7.980477783
704	12-Hydroxychelirubine	$C_{21}H_{17}NO_6$	C05193	759.2156	3.741783098
705	Flavonol base + 4O, O-Hex-dHex-Pen	$C_{32}H_{38}O_{20}$	C10175	760.2281	1.789

706	PC(16_0_18_1(9Z))	$C_{42}H_{82}NO_8P$	C13875	760.5763	11.50524951
707	omega-Hydroxy-beta-dihydromenaquinone-9	$C_{56}H_{82}O_3$	C21885	786.6099	3.572774637
708	Podofilox	$C_{22}H_{22}O_8$	C10874	829.2637	1.44563477
709	Amylopectin	$C_{30}H_{52}O_{26}$	C00317	829.2919	12.00346935
710	Verbascose	$C_{30}H_{52}O_{26}$	C08252	829.269	5.12
711	alpha-Chaconine	$C_{45}H_{73}NO_{14}$	C10796	852.5177	8.614
712	alpha-Solanine	$C_{45}H_{73}NO_{15}$	C10820	868.5149	11.087

Table S5. KEGG metabolite components annotated in the negative ion mode

Number	Metabolite	Formula	KEGG Compound ID	M/Z	ppm
1	L-Erythrulose	$C_4H_8O_4$	C02022	101.0232	12.031
2	2-Methyl-3-oxopropanoic acid	$C_4H_6O_3$	C00349	101.0232	12.038
3	(r)-Methylmalonate semialdehyde	$C_4H_6O_3$	C21030	101.0231	12.038
4	4-Aminoisoxazolidin-3-one	$C_3H_6N_2O_2$	C06682	101.0232	12.038
5	(S)-Methylmalonic acid semialdehyde	$C_4H_6O_3$	C06002	101.0229	15.008
6	Alpha-ketobutyrate	$C_4H_6O_3$	C00109	101.0232	12.038
7	4-Aminophenol	C_6H_7NO	C02372	108.0444	10.04
8	Hydroquinone	$C_6H_6O_2$	C00530	109.0286	8.265
9	Methyl sulfate	CH_4O_4S	C02704	111.0187	11.709
10	2-Furoic acid	$C_5H_4O_3$	C01546	111.0077	9.612
11	Creatinine	$C_4H_7N_3O$	C00791	112.0501	13.679
12	5,6-dihydrouracil	$C_4H_6N_2O_2$	C00429	113.0239	4.567
13	2-Deoxy-L-ribono-1,4-lactone	$C_5H_8O_4$	C02674	113.0238	4.56
14	Oxopent-4-enoate	$C_5H_6O_3$	C00596	113.0238	5.452
15	3-Hydroxycyclohexanone	$C_6H_{10}O_2$	C03228	113.0238	5.452
16	Acetylenedicarboxylic acid	$C_4H_2O_4$	C03248	112.9858	19.761
17	4-Hydroxy-2-oxovalerate	$C_5H_8O_4$	C03589	113.0231	6.329

18	epsilon-Caprolactone	$C_6H_{10}O_2$	C01880	113.023	12.53
19	N-Methylhydantoin	$C_4H_6N_2O_2$	C02565	113.0597	9.726
20	L-3-Cyanoalanine	$C_4H_6N_2O_2$	C02512	113.0238	5.452
21	3-Butyn-1-al	C_4H_4O	C06145	113.0239	4.574
22	Isobutyronitrile	C_4H_7N	C02420	114.0549	10.081
23	1-Pyrroline	C_4H_7N	C15668	114.0547	10.958
24	Glutarate semialdehyde	$C_5H_8O_3$	C03273	115.0391	6.653
25	FA 5_0;O2	$C_5H_{10}O_4$	C04272	115.039	8.385
26	Dihydropteridine	$C_6H_6N_4$	C05649	115.0393	6.646
27	Propynoic acid	$C_3H_2O_2$	C00804	115.0026	9.415
28	Aspartate semialdehyde	$C_4H_7NO_3$	C00441	116.0701	13.769
29	5-Aminopentanoic acid	$C_5H_{11}NO_2$	C00431	116.0714	2.569
30	(Z)-2-methyl-peroxyaminoacrylate	$C_4H_7NO_3$	C20250	116.0347	5.298
31	Indole	C_8H_7N	C00463	116.0701	13.769
32	Hypoxanthine	$C_5H_4N_4O$	C00262	117.018	19.381
33	Acrolein	C_3H_4O	C01471	117.0186	4.546106267
34	Iron	Fe	C14819	116.9275	4.769
35	Mesoxalic acid	$C_3H_2O_5$	C00830	117.018	11.376
36	Succinic acid	$C_4H_6O_4$	C00042	117.0179	12.23
37	5-Hydroxypentanoic acid	$C_5H_{10}O_3$	C02804	117.0546	9.522
38	Threonic acid	$C_4H_8O_5$	C01620	117.0179	11.369
39	Methyl acetate	$C_3H_6O_2$	C17530	119.0339	9.083
40	3-Hydroxypropanal	$C_3H_6O_2$	C00969	119.0341	7.402751817
41	Propanal	C_3H_6O	C00479	119.0337	7.403
42	Erythrose	$C_4H_8O_4$	C01796	119.0341	7.396
43	(S)-1,2-Epoxypropane	C_3H_6O	C11507	119.0341	7.402751817
44	Lactaldehyde	$C_3H_6O_2$	C00424	119.0341	7.403
45	Hydroxyacetone	$C_3H_6O_2$	C05235	119.0337	10.763
46	Dimethylaniline-N-oxide	$C_8H_{11}NO$	C01183	119.0492	8.687
47	3-Hydroxybenzaldeh	$C_7H_6O_2$	C03067	121.0282	10.751

	yde				
48	2-Hydroxybenzaldehyde	$C_7H_6O_2$	C06202	121.0282	10.75104728
49	4-Hydroxymethylcatechol	$C_7H_8O_3$	C14110	121.0282	10.744
50	5-(Hydroxymethyl)-2-methylpyrimidin-4-OL	$C_6H_8N_2O_2$	C76221	122.0232	0.404671349
51	1,4,5,6-Tetrahydro-6-oxonicotinic acid	$C_6H_7NO_3$	C04226	122.023	1.227
52	Nicotinic acid	$C_6H_5NO_2$	C00253	122.0231	13.527
53	3-hydroxybenzyl alcohol	$C_7H_8O_2$	C03351	123.0441	8.537
54	Aminohydroquinone	$C_6H_7NO_2$	C14604	124.0384	16.122
55	Pyridine	C_5H_5N	C00747	124.0385	15.322
56	Sumiki's acid	$C_6H_6O_4$	C20448	124.0063	18.425
57	3-hydroxyaminophenol	$C_6H_7NO_2$	C14602	124.0386	14.51
58	3-Ethyl-1,2-cyclopentanedione	$C_7H_{10}O_2$	C00037	125.0228	8.928
59	Triacetic acid	$C_6H_8O_4$	C01757	125.0233	8.921
60	4-hydroxy-5-methyl-2-methylene-3(2H)-furanone	$C_6H_6O_3$	C20718	125.0231	10.527
61	5-Hydroxymethyl-2-furancarboxaldehyde	$C_6H_6O_3$	C11101	125.0239	4.129
62	2-Hydroxyethylphosphonate	$C_2H_7O_4P$	C06451	125.0234	8.128
63	1,2,3-Trihydroxybenzene	$C_6H_6O_3$	C01108	125.0232	9.728
64	triacetate lactone	$C_6H_6O_3$	C02752	125.0233	11.327
65	2-Oxo-4-hydroxy-5-aminovalerate	$C_5H_9NO_4$	C05941	128.0333	8.701

66	Strombine	$C_5H_9NO_4$	C03790	128.0333	15.73
67	(2E)-Decenoyl-ACP	$C_6H_{11}NO_2$	C03969	128.0715	1.547
68	Pyrroline hydroxycarboxylic acid	$C_5H_7NO_3$	C04281	128.0335	14.174
69	Pyroglutamic acid	$C_5H_7NO_3$	C01879	128.0721	3.137
70	L-4-Hydroxyglutamate semialdehyde	$C_5H_9NO_4$	C05938	128.0332	16.511
71	4-Oxoproline	$C_5H_7NO_3$	C01877	128.0332	16.517
72	Adipate semialdehyde	$C_6H_{10}O_3$	C06102	129.0539	14.06
73	D-threo-3-Methylmalate	$C_5H_8O_5$	C06031	129.0178	9.536
74	Ribonolactone	$C_5H_8O_5$	C00652	129.0178	11.862
75	Isopentyl acetate	$C_7H_{14}O_2$	C12296	129.0539	14.06
76	2-Methyl-2-vinyloxirane	C_5H_8O	C21373	129.0539	14.06683487
77	Citraconic acid	$C_5H_6O_4$	C02226	129.0178	11.868
78	Ketoleucine	$C_6H_{10}O_3$	C00233	129.0178	11.868
79	5-methylthiopentanal doxime	$C_6H_{13}NOS$	C17245	129.0377	0.345790388
80	dihydro-3-hydroxy-4,4-dimethyl- 2(3H)-Furanone	$C_6H_{10}O_3$	C01012	129.0547	7.862
81	chloric acid	$ClHO_3$	C01485	128.9586	7.957436728
82	D-erythro-3-Methylmalate	$C_5H_8O_5$	C06032	129.0178	11.862
83	Cyclopentanone	C_5H_8O	C00557	129.0542	14.067
84	3-methylmalate(2-)	$C_5H_8O_5$	C06029	129.0178	11.862
85	Trans-4-hydroxyproline	$C_5H_9NO_3$	C01157	130.0484	17.408
86	5-Amino-2-oxopentanoic acid	$C_5H_9NO_3$	C01110	130.0485	19.715
87	5-Aminolevulinic acid	$C_5H_9NO_3$	C00430	130.0487	17.408
88	L-Norleucine	$C_6H_{13}NO_2$	C01933	130.0848	19.582
89	Piperidine	$C_5H_{11}N$	C01746	130.0848	19.588
90	Creatine	$C_4H_9N_3O_2$	C00300	130.0489	15.871
91	L-Isoleucine	$C_6H_{13}NO_2$	C00407	130.0485	18.946

92	L-Arabinose	$C_5H_{10}O_5$	C02479	131.034	7.475768884
93	2-Butyne-1,4-diol	$C_4H_6O_2$	C02497	131.0337	7.488
94	D-Ribulose	$C_5H_{10}O_5$	C00309	131.0339	8.239
95	6-Hydroxyhexanoic acid	$C_6H_{12}O_3$	C06103	131.0707	5.064
96	Acetoin	$C_4H_8O_2$	C00466	133.049	12.254
97	Indan-1-ol	$C_9H_{10}O$	C01710	133.0649	7.399
98	beta-Alaninamide	$C_3H_8N_2O$	C19779	133.0491	11.502
99	DL-Malic acid	$C_4H_6O_5$	C00149	133.0132	7.865
100	(R)-Acetoin	$C_4H_8O_2$	C00810	133.0494	9.247
101	D-Arabitol	$C_5H_{12}O_5$	C01904	133.049	12.242
102	3-Sulfinoalanine	$C_3H_7NO_4S$	C00606	133.9908	6.991
103	Propanoyl phosphate	$C_3H_7O_5P$	C02876	135.97	5.260131647
104	Salicylic acid	$C_7H_6O_3$	C00805	137.0233	8.146
105	2-Nitrophenol	$C_6H_5NO_3$	C01988	138.0186	7.72
106	Dimethyl sulfoxide	C_2H_6OS	C11143	139.0386	10.545
107	FA 6_3;O2	$C_6H_6O_4$	C02480	141.0185	5.894
108	Imidazolone acetate	$C_5H_6N_2O_3$	C05133	141.0296	6.837
109	O-acetylhomoserine	$C_6H_{11}NO_4$	C01077	142.0503	4.669
110	2-Deoxy-3-keto-scyllo-inosamine	$C_6H_{11}NO_4$	C17581	142.0499	7.484
111	2-methyleneglutaric acid	$C_6H_8O_4$	C02930	143.0334	0.836
112	Methylitaconate	$C_6H_8O_4$	C02295	143.0352	2.235
113	ascopyrone M	$C_6H_8O_4$	C19822	143.0342	0.562
114	(±)-2,2'-Iminobispropanoic acid	$C_6H_{11}NO_4$	C03210	143.0348	1.253
115	Aminoadipic acid	$C_6H_{11}NO_4$	C00956	143.0334	11.041
116	4-Acetamidobutanoate	$C_6H_{11}NO_3$	C02946	144.0655	7.727
117	3-Methyleneoxindole	C_9H_7NO	C02796	144.0655	7.033
118	(2R)-2-Hydroxy-2-methylbutanenitrile	C_5H_9NO	C18796	144.0656	7.038
119	Methylpyrrolidone	C_5H_9NO	C11118	144.0656	7.038320737
120	Indole-3-carboxaldehyde	C_9H_7NO	C08493	144.0453	1.283
121	2-Amino-5-	$C_6H_{11}NO_3$	C05825	144.0654	8.421

	oxohexanoate				
122	quinolone	C_9H_7NO	C06338	144.0655	7.033
123	2-Deoxy-scyllo-inosamine	$C_6H_{13}NO_4$	C17580	144.0656	7.027
124	4-Hydroxyquinoline	C_9H_7NO	C06343	144.0436	13.085
125	Allysine	$C_6H_{11}NO_3$	C04076	144.0656	7.033
126	Oxoglutaric acid	$C_5H_6O_5$	C00026	145.0133	6.525
127	P-Coumaraldehyde	$C_9H_8O_2$	C05608	147.029	6.09
128	D-Xylonic acid	$C_5H_{10}O_6$	C00502	147.029	6.084
129	4-Methylthiobutylthiohydroximate	$C_5H_{11}NOS_2$	C17243	146.995	4.230212592
130	1-Methylxanthine	$C_6H_6N_4O_2$	C16358	147.0288	16.542
131	Homovanillin	$C_9H_{10}O_3$	C05581	147.0442	6.458
132	oxfenicine	$C_8H_9NO_3$	C12323	148.0414	6.762
133	prenyl phosphate	$C_5H_{11}O_4P$	C21214	147.9621	15.753
134	D-Apiose	$C_5H_{10}O_5$	C01488	149.0447	5.667
135	N-(Acetyloxy)benzenamine	$C_8H_9NO_2$	C02709	150.0545	10.323
136	6-Methylsalicylic acid	$C_8H_8O_3$	C02657	151.039	7.054
137	Mandelic acid	$C_8H_8O_3$	C01984	151.0394	4.405
138	Xanthine	$C_5H_4N_4O_2$	C00385	151.0253	5.614820166
139	3,4-Dihydroxyphenylacetaldehyde	$C_8H_8O_3$	C04043	151.0393	5.067
140	Glycerate	$C_3H_6O_4$	C00258	151.0252	2.575
141	3-Amino-5-hydroxybenzoic acid	$C_7H_7NO_3$	C12107	152.0346	4.701
142	m-Cresol	C_7H_8O	C01467	153.0549	5.327368807
143	2,6-Dihydroxybenzoic acid	$C_7H_6O_4$	C21298	153.0186	4.778
144	1,2-Benzoquinone	$C_6H_4O_2$	C02351	153.0186	4.784
145	Gentisate	$C_7H_6O_4$	C00628	153.0186	4.778
146	cis-1,2-Dihydroxycyclohexa-3,5-diene-1-carboxylate	$C_7H_8O_4$	C06321	155.0343	4.389

147	CYCLOHEXANECARBOXYLIC ACID, 4-HYDROXY-3-OXO- (9CI)	$C_7H_{10}O_4$	C04670	157.0489	11.013
148	5-Acetamidovalerate	$C_7H_{13}NO_3$	C03087	158.0811	7.353
149	3-Dehydrocarnitine	$C_7H_{13}NO_3$	C02636	158.0809	8.618244835
150	N-Norgramine	$C_{10}H_{12}N_2$	C21602	158.9778	1.744
151	Lactyl-Brenztraubensaure	$C_6H_8O_5$	C20781	159.1024	1.648
152	Tryptamine	$C_{10}H_{12}N_2$	C00398	159.0288	6.888
153	3,5_4-Trihydroxycyclohexa-1,2-dione	$C_6H_8O_5$	C04287	159.0291	5.001
154	2,4-DICHLOROTOLUENE	$C_7H_6Cl_2$	C18300	158.9779	3.288633829
155	Quinoline-3,4-diol	$C_9H_7NO_2$	C11503	160.0401	1.873
156	Shikomol	$C_{10}H_{10}O_2$	C10472	161.0599	5.585
157	D-1,5-Anhydrofructose	$C_6H_{10}O_5$	C06485	161.0428	17.042
158	2-dehydro-3-deoxy-L-rhamnonate	$C_6H_{10}O_5$	C03979	161.0427	17.663
159	2-Deoxyinosose	$C_6H_{10}O_5$	C17209	161.0464	5.312
160	Propioin	$C_6H_{12}O_2$	C02948	161.0812	4.524
161	(S)-3-Amino-3-phenylpropanoic acid	$C_9H_{11}NO_2$	C20488	164.0722	1.23
162	L-Phenylalanine	$C_9H_{11}NO_2$	C00079	164.0719	1.23
163	Methylnoradrenaline	$C_9H_{13}NO_3$	C17925	164.0719	1.235
164	7-hydroxy-4-isopropenyl-7-methyloxepan-2-one	$C_{10}H_{16}O_3$	C19079	165.0548	11.595
165	Choline	$[C_5H_{14}NO]^+$	C00114	165.1026	11.79884569
166	L-3-Phenyllactic acid	$C_9H_{10}O_3$	C05607	165.0547	6.147
167	Se-Methylselenocysteine	$C_4H_9NO_2Se$	C05689	164.9455	3.158

168	Purine	$C_5H_4N_4$	C15587	165.0389	17.559
169	3,4-Dihydroxymandelic acid	$C_8H_8O_5$	C05580	165.0186	4.426045912
170	bornane-2,6-dione	$C_{10}H_{14}O_2$	C20320	165.0762	3.895
171	Styrene	C_8H_8	C07083	165.0548	5.545915054
172	2,6-Dihydroxyphenylacetate	$C_8H_8O_4$	C06207	167.0345	2.876
173	Pyridoxamine	$C_8H_{12}N_2O_2$	C00534	167.0819	4.170289541
174	4-hydroxymandelic acid	$C_8H_8O_4$	C11527	167.0345	2.876
175	Pyridoxine	$C_8H_{11}NO_3$	C00314	168.0663	1.863
176	Norepinephrine	$C_8H_{11}NO_3$	C00547	168.0664	1.268
177	N-Acetylleucine	$C_8H_{15}NO_3$	C02710	172.0968	6.459
178	Aconitate [cis or trans]	$C_6H_6O_6$	C00417	173.0084	4.4
179	Cyanuric acid	$C_3H_3N_3O_3$	C06554	174.0149	3.703013533
180	Ascorbate radical	$C_6H_7O_6$	C01041	174.015	11.9862144
181	4-Methylumbelliferone	$C_{10}H_8O_3$	C03081	175.0394	3.801
182	D-Glucurono-6,3-lactone	$C_6H_8O_6$	C02670	175.0242	3.487
183	2-Isopropylmalic acid	$C_7H_{12}O_5$	C02504	175.0604	4.534
184	Tagaturonate	$C_6H_{10}O_7$	C00558	176.0109	11.786
185	Stilbene oxide	$C_{14}H_{12}O$	C16014	177.0407	1.927
186	2-Inosose	$C_6H_{10}O_6$	C00691	177.0408	1.923
187	2-Keto-3-deoxy-D-gluconic acid	$C_6H_{10}O_6$	C00204	177.0408	0.793
188	1,7-dimethylurate	$C_7H_8N_4O_3$	C16356	177.0408	5.627967677
189	Daphnetol	$C_9H_6O_4$	C03093	177.0187	3.566
190	3,6-anhydro-L-galactonic acid	$C_6H_{10}O_6$	C20903	177.0407	0.793
191	L-galactono-1,5-lactone	$C_6H_{10}O_6$	C21955	177.0406	0.793
192	Altronic acid	$C_6H_{12}O_7$	C00817	177.0406	0.798
193	D-Gulono-1,4-lactone	$C_6H_{10}O_6$	C05410	177.0406	0.228
194	2D-2,3,5_4,6-	$C_6H_{10}O_6$	C20251	177.0388	0.793

	pentahydroxycyclohexanone				
195	Tetrahydroxypteridine	$C_6H_4N_4O_4$	C03178	176.8736	6.888
196	Pyrophosphate	$H_4O_7P_2$	C00013	176.9352	4.256
197	D-Galactonolactone	$C_6H_{10}O_6$	C02669	177.0394	5.985
198	Aspirin	$C_9H_8O_4$	C01405	179.0346	2.125
199	Laevuflex	$C_6H_{12}O_6$	C00247	179.0529	17.921
200	D-Aldose	$C_6H_{12}O_6$	C00737	179.0528	18.479
201	4-Hydroxyphenylpyruvic acid	$C_9H_8O_4$	C01179	179.0348	1.007513065
202	D-Tagatose	$C_6H_{12}O_6$	C00795	179.0524	12.089
203	4-Methylbenzoic acid	$C_8H_8O_2$	C01454	181.05	0.385
204	Galactitol	$C_6H_{14}O_6$	C01697	181.071	4.186
205	Pyridoxate	$C_8H_9NO_4$	C00847	182.0453	3.18
206	Sebacic acid	$C_{10}H_{18}O_4$	C08277	183.102	3.612
207	Bergaptol	$C_{11}H_6O_4$	C00758	183.0111	12.7523418
208	Panthenol	$C_9H_{19}NO_4$	C05944	186.1128	4.083
209	2-Keto-6-acetamidocaproate	$C_8H_{13}NO_4$	C05548	186.0762	5.253
210	FA 8_1	$C_8H_{14}O_2$	C16654	187.097	3.089
211	FA 7_3;O4	$C_7H_8O_6$	C04002	187.0241	3.798
212	Glycylleucine	$C_8H_{16}N_2O_3$	C02155	187.0972	2.016
213	Nα-Acetyl-L-lysine	$C_8H_{16}N_2O_3$	C02727	187.1084	2.192
214	Scopoletin	$C_{10}H_8O_4$	C01752	191.0357	3.767
215	Diketogulonic acid	$C_6H_8O_7$	C04575	191.0188	4.844
216	D-glucaro-1,5-lactone	$C_6H_8O_7$	C20890	191.0551	5.28
217	2-Hydroxychromene-2-carboxylate	$C_{10}H_8O_4$	C06204	191.0708	2.951
218	Cuminaldehyde	$C_{10}H_{12}O$	C06577	193.0863	3.696688476
219	1-Methoxy-4-(2-propenyl)benzene	$C_{10}H_{12}O$	C10452	193.0864	3.178783695
220	Oxaloglycolate	$C_4H_4O_6$	C03459	193.0359	2.718
221	Cinnamic acid	$C_9H_8O_2$	C00423	193.0345	4.535
222	D-Glucuronate	$C_6H_{10}O_7$	C00191	193.0356	1.167764392
223	Leucodopachrome	$C_9H_9NO_4$	C05604	194.0452	3.499081142
224	Phosphate	H_3O_4P	C00009	194.946	2.653

225	2,4-DINITROANISOLE	$C_7H_6N_2O_5$	C21159	197.0217	6.627798359
226	Xylitol	$C_5H_{12}O_5$	C00379	197.0657	4.941
227	Nudifloramide	$C_7H_8N_2O_2$	C05842	197.045	2.768
228	Yucron	$C_6H_{14}ClNO_2S$	C04078	198.0322	3.164
229	Dodecanoic acid	$C_{12}H_{24}O_2$	C02679	199.17	1.727
230	4-Methylumbelliferyl acetate	$C_{12}H_{10}O_4$	C03837	200.0712	2.484
231	2,2,3-trihydroxybiphenyl	$C_{12}H_{10}O_3$	C03569	201.0545	6.041048074
232	L-Tryptophan	$C_{11}H_{12}N_2O_2$	C00078	203.082	0.016
233	beta-N-Acetylglucosamine	$C_8H_{15}NO_6$	C03878	203.0542	9.394437052
234	1-Naphthol	$C_{10}H_8O$	C11714	205.0502	2.098900172
235	4-Chlorocatechol	$C_6H_5ClO_2$	C02375	205.0341	6.22
236	N-Acetyl-L-phenylalanine	$C_{11}H_{13}NO_3$	C03519	206.0819	2.729
237	3-hydroxy-3-methylglutarate	$C_6H_{10}O_5$	C03761	207.0482	13.642
238	1,2-Dihydronaphthalene-1,2-diol	$C_{10}H_{10}O_2$	C04314	207.0658	2.316
239	2,4-Dichlorophenol	$C_6H_4Cl_2O$	C02625	207.0482	13.642
240	2-Dehydro-3-deoxy-D-fuconate	$C_6H_{10}O_5$	C06159	207.0481	14.125
241	Umbelliferone	$C_9H_6O_3$	C09315	207.0701	18.45
242	Formyl-5-hydroxykynurenamine	$C_{10}H_{12}N_2O_3$	C05647	207.0482	17.827
243	2-Naphthalenesulfonic acid	$C_{10}H_8O_3S$	C16202	207.0115	3.079
244	Dihydrolipoate	$C_8H_{16}O_2S_2$	C02147	207.0483	17.344
245	L-Kynurenine	$C_{10}H_{12}N_2O_3$	C00328	207.077	1.505621575
246	1-Naphthalenesulfonic acid	$C_{10}H_8O_3S$	C16201	207.0114	3.562
247	(S)-2-(Hydroxymethyl)glutarate	$C_6H_{10}O_5$	C16390	207.0494	7.846

248	FA 6_1;O3	$C_6H_{10}O_5$	C02360	207.0478	13.642
249	O-ureido-L-serine	$C_4H_9N_3O_4$	C20639	208.0539	15.895
250	Betalamic acid	$C_9H_9NO_5$	C08538	210.0762	4.652
251	5,6-Dihydroxyindole	$C_8H_7NO_2$	C05578	210.077	0.848
252	8-Methylnonenoate	$C_{10}H_{18}O_2$	C18202	215.1285	1.75
253	Methoxsalen	$C_{12}H_8O_4$	C01864	215.0309	18.975
254	Dihydroxyacetone phosphate	$C_3H_7O_6P$	C00111	214.9957	2.459490585
255	Diethylphosphate	$C_4H_{11}O_4P$	C06608	215.0323	0.986734557
256	Diethylthiophosphate	$C_4H_{11}O_3PS$	C06607	215.0126	10.48394327
257	1,2-Dihydroxy-3-methylcyclohexa-3,5-dienecarboxylate	$C_8H_{10}O_4$	C06720	215.0558	1.44
258	Pantothenic acid	$C_9H_{17}NO_5$	C00864	218.1027	3.167
259	7-Hydroxyisoflavone	$C_{15}H_{10}O_3$	C15615	219.046	3.882
260	4-Carboxy-4-hydroxy-2-oxoadipate	$C_7H_8O_8$	C04115	219.0144	1.097552946
261	3-Indoleacetaldoxime	$C_{10}H_{10}N_2O$	C02937	219.0774	0.513881852
262	Histamine	$C_5H_9N_3$	C00388	221.1544	10.805
263	3-Propylmalate	$C_7H_{12}O_5$	C02123	221.0663	1.691
264	(E)-methyl ester 3-phenyl-2-propenoic acid	$C_{10}H_{10}O_2$	C06358	223.0605	3.113863279
265	5-Acetylamino-6-formylamino-3-methyluracil	$C_8H_{10}N_4O_4$	C16365	225.0642	5.663
266	9-Fluorenone	$C_{13}H_8O$	C06712	225.0642	11.602
267	a-L-Rhamnose	$C_6H_{12}O_5$	C02476	225.0642	11.15779949
268	Theobromine	$C_7H_8N_4O_2$	C07480	225.0641	5.214603751
269	beta-L-Rhamnose	$C_6H_{12}O_5$	C02338	225.0641	11.15779949
270	1,5-Anhydro-D-mannitol	$C_6H_{12}O_5$	C07326	225.0642	11.15779949
271	alpha-L-Fucopyranose	$C_6H_{12}O_5$	C20835	225.0642	11.15779949
272	L-Rhamnulose	$C_6H_{12}O_5$	C00861	225.0641	11.15779949

273	Galactose	$C_6H_{12}O_6$	C00124	225.0599	7.504
274	Meta-Tyrosine	$C_9H_{11}NO_3$	C20807	226.0712	3.951
275	Myristic acid	$C_{14}H_{28}O_2$	C06424	227.2012	1.947
276	Germacrene A acid	$C_{15}H_{22}O_2$	C19678	233.1542	2.122
277	3-Hydroxyflavone	$C_{15}H_{10}O_3$	C01495	237.0516	17.357
278	6-Hydroxyflavanone	$C_{15}H_{12}O_3$	C14221	239.0672	0.593
279	5-Hydroxyconiferaldehyde	$C_{10}H_{10}O_4$	C12204	239.0594	13.764
280	Guanidinoethyl methyl phosphate	$C_4H_{12}N_3O_4P$	C04071	242.0512	10.922
281	Aminoparathion	$C_{10}H_{16}NO_3PS$	C06605	243.0244	2.560154042
282	2,6-Dinitrotoluene	$C_7H_6N_2O_4$	C11008	243.028	8.747
283	Pseudouridine	$C_9H_{12}N_2O_6$	C02067	243.0621	0.645
284	Glyceric acid 1,3-biphosphate	$C_3H_8O_{10}P_2$	C00236	247.9219	14.30845722
285	2,3-Diphosphoglyceric acid	$C_3H_8O_{10}P_2$	C01159	247.9219	14.308
286	Deoxyinosine	$C_{10}H_{12}N_4O_4$	C05512	251.0775	4.280669913
287	Palmitic acid	$C_{16}H_{32}O_2$	C00249	255.2327	0.943
288	5-Methylthioribose 1-phosphate	$C_6H_{13}O_7PS$	C04188	259.0074	10.48858102
289	Clavaminate	$C_8H_{10}N_2O_4$	C06660	259.0568	1.438217024
290	D-Tagatose 1-phosphate	$C_6H_{13}O_9P$	C02888	259.0219	2.09
291	3-[(1R,2S,5R,6S)-5-hydroxy-7-oxabicyclo[4.1.0]heptan-2-yl]pyruvic acid	$C_9H_{12}O_5$	C21087	261.0731	14.34
292	Oxaloacetate	$C_4H_4O_5$	C00036	263.0032	4.830283054
293	Indanone	C_9H_8O	C01504	263.077	0.902
294	Juvenile hormone III	$C_{16}H_{26}O_3$	C09694	265.1481	11.598
295	6-Acetyl-D-glucose	$C_8H_{14}O_7$	C02655	267.0724	0.925
296	2'-Hydroxydaidzein	$C_{15}H_{10}O_5$	C02495	269.0437	6.856060558
297	Sinapaldehyde	$C_{11}H_{12}O_4$	C05610	269.0672	1.955719984
298	Norlaudanosoline	$C_{16}H_{17}NO_4$	C02916	269.084	8.82
299	N-Chloroacetyl-2,6-diethylaniline	$C_{12}H_{16}ClNO$	C21712	270.0913	3.927635211

300	Conchosin A	$C_{15}H_{18}O_5$	C09363	277.1563	1.956
301	Alcophosphamide	$C_7H_{17}Cl_2N_2O_3P$	C16551	277.033	17.674
302	Linoleic acid	$C_{18}H_{32}O_2$	C01595	279.2316	4.802
303	2-oxostearic acid	$C_{18}H_{34}O_3$	C00869	279.2335	2.006
304	2,3,5-Trihydroxytoluene	$C_7H_8O_3$	C03338	279.0881	2.482442283
305	Dehydrocoformycin	$C_{11}H_{14}N_4O_5$	C02243	281.0877	0.361
306	Rhein	$C_{15}H_8O_6$	C10401	283.2652	3.392
307	11beta-OHA4	$C_{19}H_{26}O_3$	C05284	283.1708	1.613
308	(R)-5-Diphosphomevalonic acid	$C_6H_{14}O_{10}P_2$	C01143	288.987	4.772
309	D-Sedoheptulose 7-phosphate	$C_7H_{15}O_{10}P$	C20956	289.0335	1.711
310	Gingerol	$C_{17}H_{26}O_4$	C10462	293.1761	0.944
311	9-Oxo-ODE	$C_{18}H_{30}O_3$	C14766	293.2124	0.66
312	3-HODE + 9-HODE	$C_{18}H_{32}O_3$	C14762	295.2277	0.528
313	dapdiamide C	$C_{13}H_{22}N_4O_5$	C20964	295.1375	12.43752454
314	Capsidiol	$C_{15}H_{24}O_2$	C09627	297.1711	1.214856021
315	Gibberellin A9	$C_{19}H_{24}O_4$	C11863	297.1529	11.075
316	Ricinoleic acid	$C_{18}H_{34}O_3$	C08365	297.2433	0.69
317	FA 18_1;O	$C_{18}H_{34}O_3$	C19418	297.2436	0.32
318	3,4-Dihydroxy-9,10-secoandrosta-1,3,5(10)-triene-9,17-dione	$C_{19}H_{24}O_4$	C04793	297.1529	11.075
319	FA 18_0;O2	$C_{18}H_{36}O_4$	C15988	297.1529	11.075
320	Picein	$C_{14}H_{18}O_7$	C10720	297.1529	11.073
321	FA 18_0;O	$C_{18}H_{36}O_3$	C03195	299.26	2.826
322	all-trans-Retinoic acid	$C_{20}H_{28}O_2$	C00777	299.1605	15.908
323	Pratensein	$C_{16}H_{12}O_6$	C10520	299.2009	2.481
324	Eicosapentaenoic acid	$C_{20}H_{30}O_2$	C06428	301.217	1.632
325	ent-3beta-hydroxycassa-12,15-dien-2-one	$C_{20}H_{30}O_2$	C21826	301.2174	0.359939698
326	Cytidine monophosphate	$C_9H_{14}N_3O_8P$	C00055	305.0212	10.4124569
327	N-	$C_{11}H_{19}NO_9$	C00270	308.099	0.973778234

	acetylneuraminate				
328	ACMC-20m5rp	$C_{18}H_{30}O_4$	C20704	309.2071	0.07
329	corytuberine	$C_{19}H_{21}NO_4$	C17591	309.1193	19.652
330	trans-cinnamoyl-beta-D-glucoside	$C_{15}H_{18}O_7$	C04164	309.1012	10.12167249
331	N-Methylethanolaminium phosphate	$C_3H_{10}NO_4P$	C01210	309.062	0.678116041
332	ST 21_4;O2	$C_{21}H_{28}O_2$	C03207	311.2236	2.664
333	9Z-Octadecenedioic acid	$C_{18}H_{32}O_4$	C19618	311.2232	1.379
334	methyl gibberellin A9	$C_{20}H_{26}O_4$	C20632	311.169	12.02504138
335	Sanguinarine	$[C_{20}H_{14}NO_4]^+$	C06162	313.0787	13.617
336	Floionolic acid	$C_{18}H_{36}O_5$	C19621	314.1689	12.658
337	3-Ketosucrose	$C_{12}H_{20}O_{11}$	C05731	321.0829	0.574
338	Sterigmatocystin	$C_{18}H_{12}O_6$	C00961	323.0545	4.979902462
339	beta-L-Fucose	$C_6H_{12}O_5$	C20836	327.1298	0.415798561
340	Gibberellin A3	$C_{19}H_{22}O_6$	C01699	328.1844	11.663
341	Cortexolone	$C_{21}H_{30}O_4$	C05488	328.1844	11.663
342	Stearic acid	$C_{18}H_{36}O_2$	C01530	329.2707	2.979
343	Tetrahydropalmatine	$C_{21}H_{25}NO_4$	C02890	337.1439	1.849595677
344	2-Biphenylol	$C_{12}H_{10}O$	C02499	339.138	3.083051442
345	(S)-4',5,7-Trihydroxy-6-prenylflavanone	$C_{20}H_{20}O_5$	C09832	339.2297	9.553
346	Crocetindial	$C_{20}H_{24}O_2$	C19730	341.1797	11.36064103
347	10-Deoxymethynolide	$C_{17}H_{28}O_4$	C11993	341.1962	2.205123914
348	1-O-Caffeoyl-beta-D-glucose	$C_{15}H_{18}O_9$	C10433	341.0864	4.108577475
349	Chrysosplenol	$C_{18}H_{16}O_8$	C10031	341.0649	4.609618222
350	10S-HpOME	$C_{18}H_{34}O_4$	C20702	359.0831	16.325
351	Fluconazole	$C_{13}H_{12}F_2N_6O$	C07002	367.1036	16.401
352	Cortol	$C_{21}H_{36}O_5$	C05482	367.2456	9.215
353	Carboprost Tromethamine	$C_{21}H_{36}O_5$	C06872	367.2464	2.766
354	Averufin	$C_{20}H_{16}O_7$	C20574	367.246	8.126
355	Aflatoxin G2	$C_{17}H_{14}O_7$	C16754	375.0699	6.006826727
356	Chamuvarin	$C_{23}H_{22}O_5$	C09759	377.0823	2.715

357	aurachin C epoxide	$C_{25}H_{33}NO_3$	C21331	377.2177	14.56617783
358	Indole-3-acetonitrile-glycylcysteine conjugate	$C_{15}H_{16}N_4O_3S$	C21665	377.0917	2.148
359	Patuletin	$C_{16}H_{12}O_8$	C10118	377.0835	11.165
360	Carbenicillin	$C_{17}H_{18}N_2O_6S$	C06869	377.0666	9.132
361	Diphyllin	$C_{21}H_{16}O_7$	C10559	379.0821	0.586099686
362	N-Formyldemecolcine	$C_{22}H_{25}NO_6$	C16710	380.0841	10.271
363	Macrozamin	$C_{13}H_{24}N_2O_{11}$	C08504	383.119	2.333
364	NSC-14980	$C_{12}H_{20}O_{11}$	C01093	385.1001	3.478
365	(1S,5R)-5-Hydroxyaverantin	$C_{20}H_{20}O_8$	C20501	387.1142	14.633
366	Triamcinolone	$C_{21}H_{27}FO_6$	C07158	393.1715	0.969
367	Legumelin	$C_{23}H_{22}O_6$	C10417	393.1714	1.223
368	isopentenyl adenosine	$C_{15}H_{21}N_5O_4$	C16427	396.1467	14.55314382
369	80666-03-1	$C_{22}H_{25}NO_7$	C20297	396.145	0.637594568
370	PC-M6	$C_{27}H_{35}NO_3$	C20535	420.2486	13.80987325
371	Talatizamine	$C_{24}H_{39}NO_5$	C08713	420.2602	13.793
372	alpha-Tocotrienol	$C_{29}H_{44}O_2$	C14153	423.3266	0.557
373	Desacetylmycothiol	$C_{15}H_{28}N_2O_{11}S$	C19703	425.1287	12.156
374	4-hydroxylamino-2,6-dinitrotoluene	$C_7H_7N_3O_5$	C16392	425.0705	1.50097478
375	Acetyl-maltose	$C_{14}H_{24}O_{12}$	C02130	429.1251	0.527
376	6-Aminopenicillanic acid	$C_8H_{12}N_2O_3S$	C02954	431.1012	12.164
377	Medicocarpin	$C_{22}H_{24}O_9$	C16223	431.1404	13.107
378	Flavin mononucleotide	$C_{17}H_{21}N_4O_9P$	C00061	455.1021	10.472
379	Diketol	$C_{27}H_{42}O_3$	C16509	459.3036	17.378
380	JI-20A	$C_{19}H_{39}N_5O_9$	C17704	463.232	19.297
381	Cholesterol sulfate	$C_{27}H_{46}O_4S$	C18043	465.3026	3.841
382	5β-Androstan-3α-ol-17-One Glucosiduronate	$C_{25}H_{38}O_8$	C11136	465.3403	6.232
383	linustatin	$C_{16}H_{27}NO_{11}$	C08333	470.1492	4.938389558
384	L-Formylkynurenine	$C_{11}H_{12}N_2O_4$	C02700	471.1546	5.242483253
385	C1-C9-Glycosylated	$C_{25}H_{28}O_9$	C18681	471.1695	7.328

	UWM6				
386	Bryophyllin A	$C_{26}H_{32}O_8$	C08852	471.0879	11.421
387	Dyspropterin	$C_9H_{11}N_5O_3$	C03684	473.158	14.994
388	Furcatin	$C_{20}H_{28}O_{10}$	C10458	473.1723	12.37946537
389	Rutaevin	$C_{26}H_{30}O_9$	C08779	485.1757	12.359
390	10-deacetyl-2-debenzoylbaccatin III	$C_{22}H_{32}O_9$	C11899	485.2897	2.674
391	ST 28_2;O4	$C_{28}H_{46}O_4$	C15792	491.3395	3.47
392	Sophorotriose	$C_{18}H_{32}O_{16}$	C21454	503.1584	6.659
393	Protopanaxadiol	$C_{30}H_{52}O_3$	C20715	505.33	4.583
394	3-Dehydroecdysone	$C_{27}H_{42}O_6$	C02513	507.283	12.07746552
395	ANTIBIOTIC A-31438	$C_{28}H_{50}O_{10}$	C06630	527.3168	10.88260397
396	Pachymic_acid	$C_{33}H_{52}O_5$	C17044	527.3311	12.695
397	Elwesine	$C_{16}H_{21}NO_3$	C21637	549.2999	5.288950717
398	D-Gal alpha 1->6D-Gal alpha 1->6D-Glucose	$C_{18}H_{32}O_{16}$	C05404	549.1752	14.511
399	Oxidized glutathione	$C_{20}H_{32}N_6O_{12}S_2$	C00127	593.1321	3.395162562
400	Flavosativaside	$C_{27}H_{30}O_{15}$	C04024	593.1453	9.924
401	82475-09-0	$C_{20}H_{31}N_3O_{11}S_2$	C20814	598.1338	7.351
402	(20R)-Ginsenoside Rh2	$C_{36}H_{62}O_8$	C22128	604.4716	9.782
403	Protoporphyrinogen IX	$C_{34}H_{40}N_4O_4$	C01079	629.305	11.03029548
404	(S)-Boldine	$C_{19}H_{21}NO_4$	C09365	653.2836	4.936
405	12(R)-HPETE	$C_{20}H_{32}O_4$	C14812	671.4631	15.313
406	(+)-Sesamin monocatechol	$C_{19}H_{18}O_6$	C21801	683.2134	0.016
407	alpha-D-Aldosyl beta-D-fructoside	$C_{12}H_{22}O_{11}$	C04219	683.2333	11.949
408	Maltotetraose	$C_{24}H_{42}O_{21}$	C02052	711.2271	9.91
409	Pluviatolide	$C_{20}H_{20}O_6$	C21191	711.2391	7.856
410	Maltopentaose	$C_{30}H_{52}O_{26}$	C06218	827.2757	10.042
411	precorrin-2	$C_{42}H_{48}N_4O_{16}$	C02463	845.2958	8.429
412	Citronellyl-CoA	$C_{31}H_{52}N_7O_{17}P_3S$	C16464	901.2109	10.444

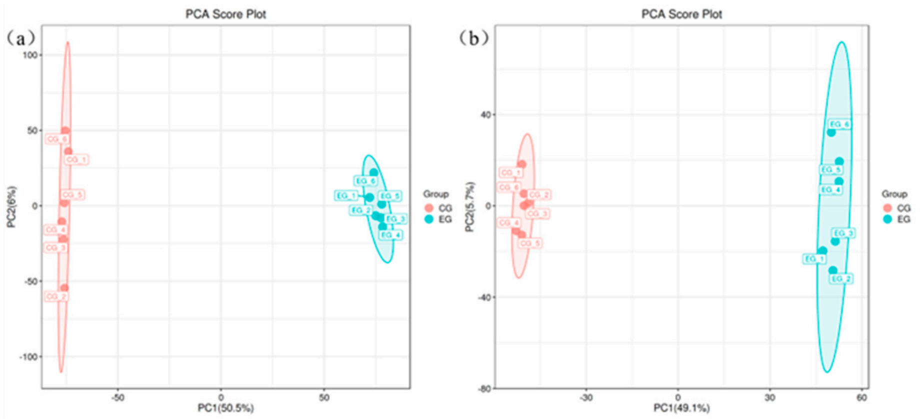

Figure S2. PCA scores of positive ion mode (a) and negative ion mode (b). PC1 represents Principal Component 1, and PC2 represents Principal Component 2. Points of the same color indicate the biological replicates within a group. The distribution pattern of the points reflects the degree of variation both between groups and within groups.

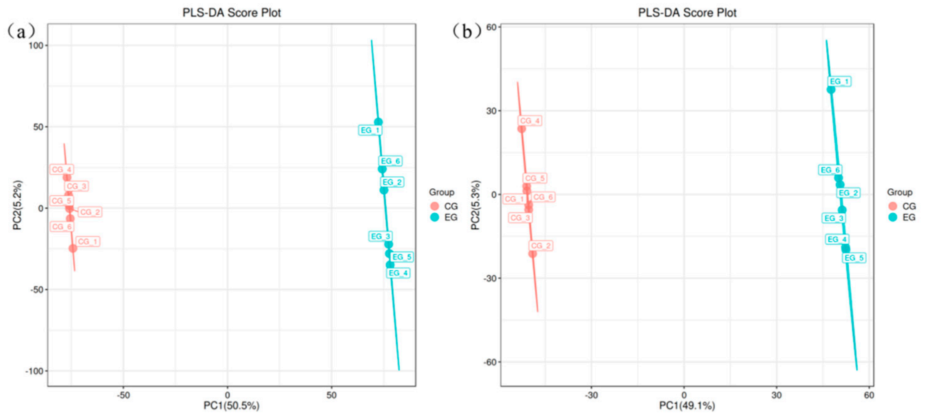

Figure S3. PLS-DA plots of metabolites in positive (a) and negative ion mode (b). PC1 represents Principal Component 1, and PC2 represents Principal Component 2. Points of the same color indicate the biological replicates within a group. The distribution pattern of the points reflects the degree of variation both between groups and within groups.

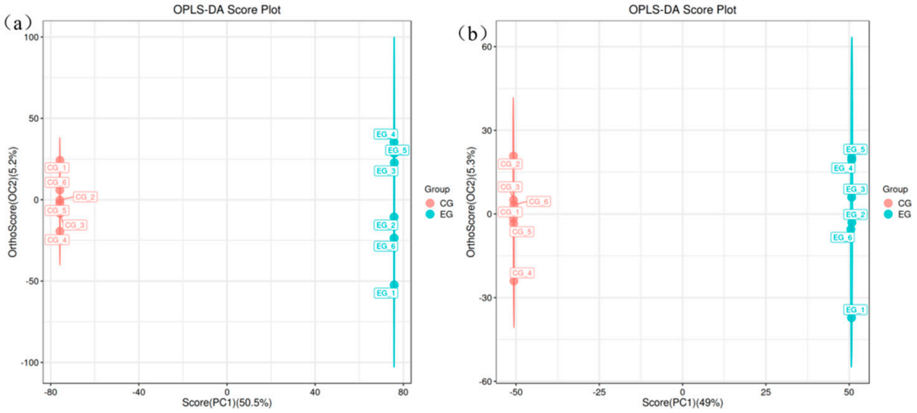

Figure S4. OPLS-DA scores in positive ion mode (a) and negative ion mode (b). PC1 represents Principal Component 1, and PC2 represents Principal Component 2. Points of the same color indicate the biological replicates within a group. The distribution pattern of the points reflects the degree of variation both between groups and within groups.

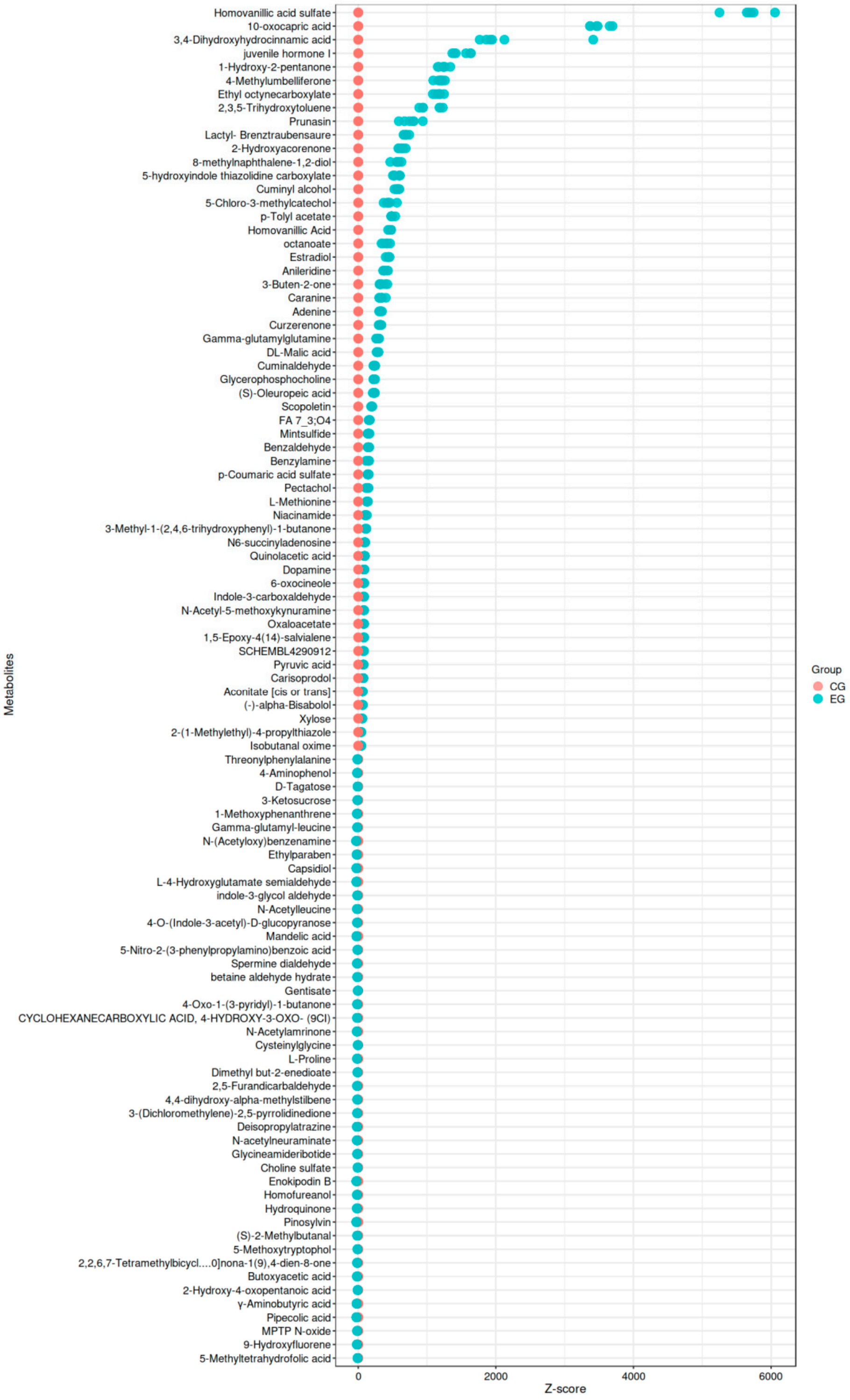

Figure S5. Z-score map of differential metabolites. Each circle in the visualization represents a sample with a Z-score >0 and <0 indicating upregulated and downregulated metabolite abundance, respectively.

Figure S6. Heatmap of correlation analysis of differential metabolites. Positive correlations (shared trends) are depicted in red, and negative correlations (divergent trends) are indicated in blue. Each dot represents the correlation between two metabolites, with darker colors and larger dots indicating stronger associations and the absence of dots signifying no correlation, The ordinate represents differential metabolites, and the abscissa represents correlation coefficients.

Table S6. Evaluation and statistics of sample sequencing data.

**Sample**	**Original sequence / piece**	**Filtered sequences / items**	**Filtering bases/G**	**Q20/%**	**Q30/%**	**GC/%**
CG-1	97,892,180	96,064,282	13,942,948,418	99.21	97.12	51.76
CG-2	56,563,490	55,424,974	8,062,037,960	99.16	96.97	51.94
CG-3	48,113,106	47,271,446	6,869,651,944	99.21	97.13	51.94
EG-1	51,055,380	50,027,066	7,272,311,635	99.16	96.96	51.82
EG-2	55,081,882	54,035,094	7,867,793,040	99.18	97.03	51.74
EG-3	56,504,068	55,729,708	8,144,919,960	99.32	97.47	51.74

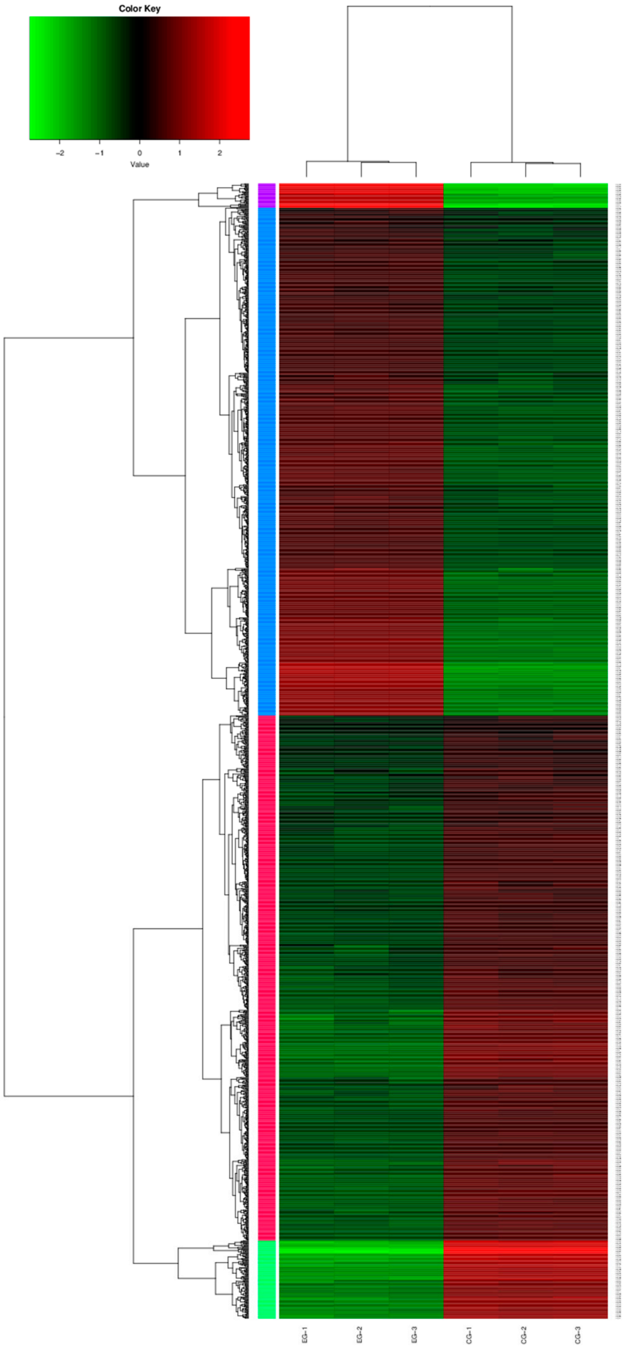

Figure S7. Differential expressions gene clustering heat map. Each column represents a sample, and each row represents a gene. Red indicates upregulation, and green indicates downregulation. On the left is the gene clustering dendrogram; the closer two gene branches are, the more similar their expression levels.

Table S7. Carbohydrate metabolic pathway number and sequence quantity information.

**Ko number**	**Pathway**	**Sequence quantity**
Ko00010	Glycolysis and gluconeogenesis	35
Ko00020	Citric acid cycle	19
Ko00030	Pentose phosphate pathway	16
Ko00040	Conversion of pentose and glucuronic acid	22
Ko00051	Fructose and mannose metabolism	17
Ko00052	Galactose metabolism	7
Ko00053	Ascorbic acid and aldehyde metabolism	10
Ko00500	Starch and sucrose metabolism	35
Ko00520	Metabolism of amino sugars and nucleotide sugars	33
Ko00562	Phosphoinositide metabolism	18
Ko00620	Pyruvate metabolism	30
Ko00630	Metabolism of glyoxylate and dicarboxylic acid	20
Ko00650	Cholesterol butyrate metabolism	12
