# Supplementary material for: Breeding of High-Polysaccharide-Producing Volvariella volvacea Strains Based on Genome Shuffling Technology
Source: J Fungi (Basel). 2025 Aug 14;11(8):591. doi: 10.3390/jof11080591 (PMC12387567; doi:10.3390/jof11080591)

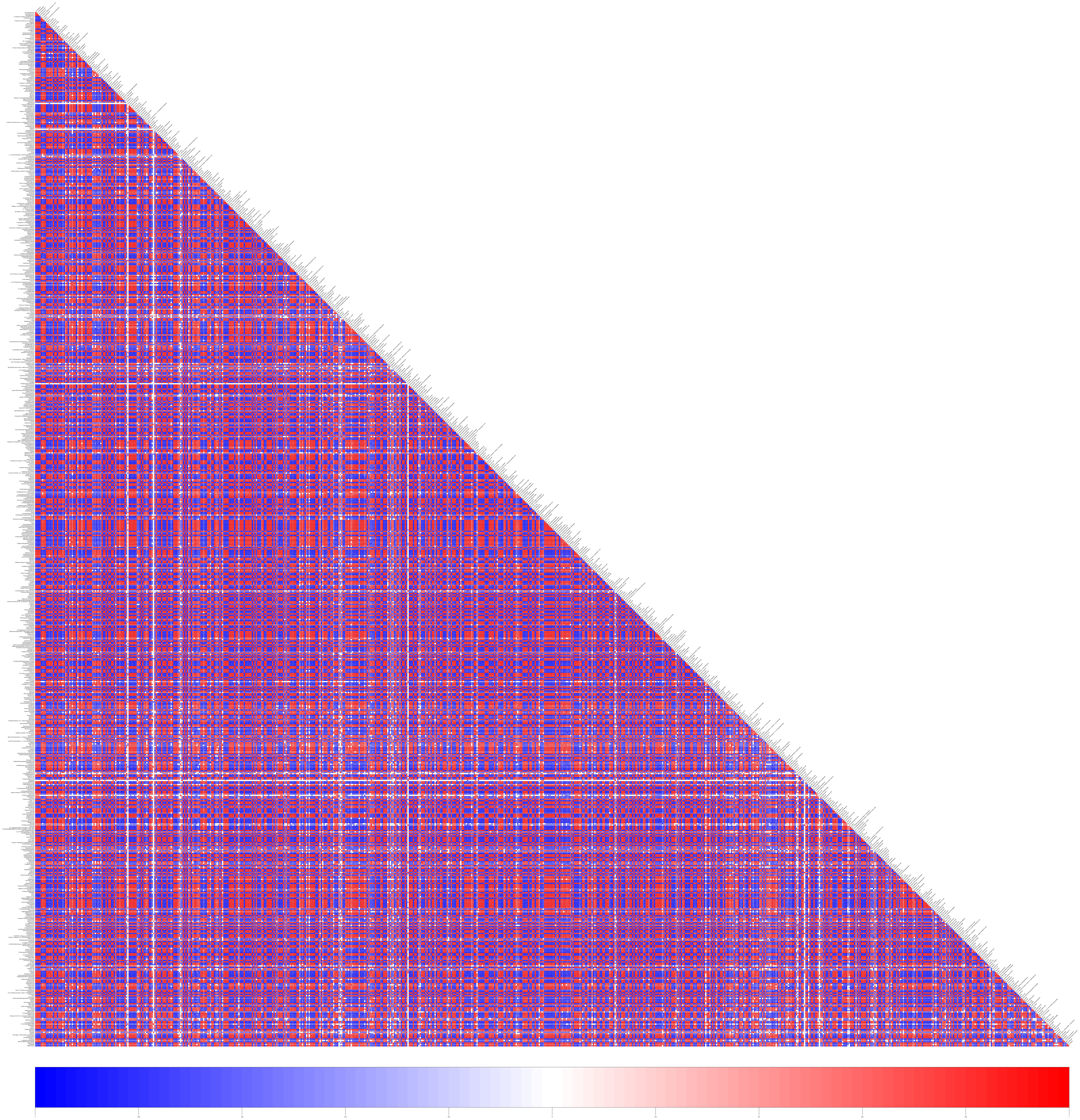

Supplement: Supplementary file 1 [file Figure S6.pdf › Figure S6.pdf]
